# Geographic Variation in Landscape Structure and Annual Climate Associations Across Cumulative Highway Buffer Scales

**DOI:** 10.3390/biology15211981

**Published:** 2026-11-02

**Authors:** Runlei Zhang, Junkai Wang, Shanfeng Wu, Wenbo Mo, Nan Yang

**Affiliations:** 1 College of Architecture & Urban Planning, Hunan City University, 518#, Yingbin Road, Yiyang 413000, China; zhangrunlei@hotmail.com (R.Z.); 15639140107@163.com (J.W.); wsf1603438805@outlook.com (S.W.); mowenbo@hncu.edu.cn (W.M.); 2 Key Laboratory of Key Technologies of Digital Urban-Rural Spatial Planning of Hunan Province, 518#, Yingbin Road, Yiyang 413000, China; 3 Key Laboratory of Urban Planning Information Technology of Hunan Provincial Universities, 518#, Yingbin Road, Yiyang 413000, China

**Keywords:** highway landscape, cumulative buffer analysis, scale dependence, land-cover composition, largest-patch dominance

## Abstract

Landscape patterns along highways vary with the spatial extent examined, and similar responses may conceal differences in land cover and patch structure. We analyzed 14 nested cumulative buffer widths (0.05–10 km) across three sections of China’s Jinggang’ao Expressway from 2000 to 2020. Generalized additive models, block bootstrap resampling, annular land-cover comparisons, and temporal regressions were used to examine landscape structure and annual climate associations. Landscape Shape Index (LSI), Shannon’s Diversity Index (SHDI), and Largest Patch Index (LPI) showed nonlinear, section-specific patterns. Initial LSI minima occurred at 0.236, 0.288, and 0.343 km, respectively, and were consistently detected under temporal resampling, although their locations depended on model specification. Despite similar LSI patterns, land-cover composition differed among sections. From 250 to 500 m, cropland proportion (PLAND) decreased by 0.94–4.36 percentage points, while the share of cropland occupied by its largest patch increased by 5.14–11.69 percentage points. Climate associations were sensitive to temporal modeling; none of 36 coefficient tests passed false discovery rate correction under autoregressive models. Combining cumulative profiles, annular composition, and class-specific patch measures reveals structural differences that aggregate landscape metrics alone may obscure.

## Simple Summary

Highways pass through landscapes made up of farmland, forests, settlements, and other land-cover types. Understanding how these landscapes differ across areas surrounding highways can help improve environmental monitoring. We examined landscape patterns at different distances from three sections of the Jinggang’ao Expressway in China between 2000 and 2020. We found that the overall landscape patterns did not change in a simple, steady way as progressively larger areas were examined. Although the three sections showed similar trends in some landscape measures, the types of land cover and the arrangement of large patches differed considerably. In particular, cropland made up a smaller share of the landscape as the areas examined became larger, even though the largest cropland patch accounted for a greater share of the cropland present. Relationships between annual climate conditions and landscape patterns also varied depending on the statistical approach used. These findings suggest that examining only one landscape measure or a single area around a highway may overlook important differences. Combining several measures can provide a clearer understanding of highway surroundings and help inform future environmental monitoring.

## 1. Introduction

Highways cross diverse landscapes, fragmenting habitats and altering ecological conditions along extensive linear corridors. Their effects can extend beyond the road surface through changes in habitat connectivity, edge environments, and human access [1,2,3,4]. However, the spatial extent of these effects varies among ecological processes and landscape settings [5]. Such variation also reflects differences in how animal groups and species respond to roads and other infrastructure [6,7]. At a broader scale, road networks contribute to the fragmentation of relatively undisturbed landscapes [8]. Roadside landscapes are also shaped by existing topography, land use, and settlement patterns. These background characteristics make it difficult to distinguish road-related effects from existing landscape patterns.

Cumulative roadside buffers provide a practical way to examine how landscape structure changes as the area surrounding a highway expands. Unlike separate distance bands, each wider cumulative buffer includes all areas covered by narrower buffers. Consequently, changes in landscape metrics across cumulative buffer widths reflect differences in the structure of progressively larger, overlapping landscapes rather than independent, distance-specific responses to highway disturbance. Landscape patterns along highways can vary across land-cover settings [9], while the sensitivity of landscape metrics to spatial scale has been widely recognized [10,11]. Three commonly used metrics—Landscape Shape Index (LSI), Shannon’s Diversity Index (SHDI), and Largest Patch Index (LPI)—describe landscape shape, land-cover diversity, and largest-patch dominance, respectively. Because these metrics capture different aspects of landscape structure [12,13,14], they may respond differently as cumulative buffer widths increase. Their interpretation also depends on the ecological questions and spatial scales being examined [15]. An important question is whether these metrics exhibit nonlinear patterns that differ among highway sections. Such patterns must be distinguished from ecological road-effect distances established through biological responses [16].

Landscape-level metrics, however, reveal only part of the structure of roadside landscapes. They do not show which land-cover classes enter the analysis as buffers widen or how patch dominance changes within individual classes. Conventional landscape metrics have limitations when used to interpret ecological patterns [17]. Habitat amount and spatial configuration are also distinct aspects of landscape structure that may have different ecological implications [18,19]. Moreover, their effects can be difficult to separate because landscape composition and configuration are often correlated [20]. Their relationships may vary with spatial scale [21,22] and over time [23]. This distinction is particularly relevant to highways passing through agricultural, forested, and developed landscapes. Two highway sections may exhibit similar cumulative LSI patterns despite substantial differences in the composition of newly included areas and in class-specific largest-patch dominance. Comparing newly added annuli with cumulative class-level metrics can help identify these structural differences without attributing LSI turning points to particular land-cover classes.

Landscape patterns may also differ along a highway as the surrounding geographic setting changes. Topography is associated with climatic and vegetation gradients [24,25], and vegetation responses to temperature and moisture conditions can vary across terrain settings [26]. Road density, accessibility, land use, and settlement patterns are also closely related [27]. Comparing different sections of the same expressway allows these geographic contrasts to be examined within a continuous corridor. In this study, LOW, MIDDLE, and HIGH refer to three geographically distinct highway sections with broadly different elevation settings, rather than elevation classes assigned to individual pixels or independent elevation treatments. Accordingly, differences among these sections are interpreted as geographic variation in landscape structure, not as effects attributable to elevation alone.

Annual climate variability adds a temporal dimension to these spatial comparisons. Temperature and moisture conditions influence vegetation growth, drought responses, and ecological recovery [28,29,30], although their associations with cumulative landscape metrics remain less clear. We use annual mean temperature (MeanTemp) and the 12-month Standardized Precipitation–Evapotranspiration Index (SPEI12), which represents climatic water-balance variability [31], to examine these relationships. Because all nested buffer widths within a highway section share the same annual climate observations, they cannot be treated as independent climate measurements [32]. Temporal trends and serial correlation can affect the interpretation of ecological time-series analyses [33], while multiple testing introduces further uncertainty when evaluating statistical associations [34]. We therefore examine two predefined annual contrasts between cumulative buffer-width groups and assess their climate associations using models that progressively account for temporal trends and first-order autoregressive [AR(1)] residual correlation. The aim is to evaluate the consistency of annual climate–landscape associations rather than infer causal climate effects or localized roadside microclimatic responses.

We investigated approximately 530 km of the Jinggang’ao Expressway in Hunan Province, China, across three geographically distinct highway sections, 14 nested cumulative buffer widths (0.05–10 km), and 21 years (2000–2020). The study addresses two landscape-structural hypotheses and one complementary analytical expectation. H1 proposes that cumulative profiles of landscape shape, diversity, and largest-patch dominance are nonlinear and metric-specific, with response characteristics differing among highway sections. H2 proposes that sections with broadly similar initial cumulative LSI response shapes may nevertheless differ in the land-cover composition of newly included roadside annuli and in class-level largest-patch dominance. H3 examines whether associations between annual MeanTemp or SPEI12 and two predefined cumulative landscape-scale contrasts remain consistent after accounting for temporal trends and residual autocorrelation. By combining cumulative landscape profiles, annular land-cover composition, class-level patch measurements, and annual climate sensitivity analyses, the study seeks to clarify how landscape structure varies along a continuous highway corridor while distinguishing these descriptive patterns from causal road or climate effects.

## 2. Materials and Methods

### 2.1. Study Area and Elevation Stratification

Hunan Province, located in central China, covers an area of approximately 211,800 km^2^. It has a humid subtropical monsoon climate and diverse topography, ranging from the Dongting Lake Plain in the north to the hilly and mountainous landscapes of central and southern Hunan [35]. The Hunan section of the Jinggang’ao Expressway (G4) extends approximately 530 km from the Hunan–Hubei border near Yanglousi (approximately 113.5° E, 29.5° N) to the Hunan–Guangdong border near Xiaotang, Yizhang County (approximately 113.0° E, 25.4° N). From north to south, the corridor passes through the Dongting Lake Plain, the Changsha–Zhuzhou–Xiangtan urban region, and the hilly and mountainous areas of southern Hunan, encompassing substantial variation in topography, land use, and human activity.

**Figure 1:**
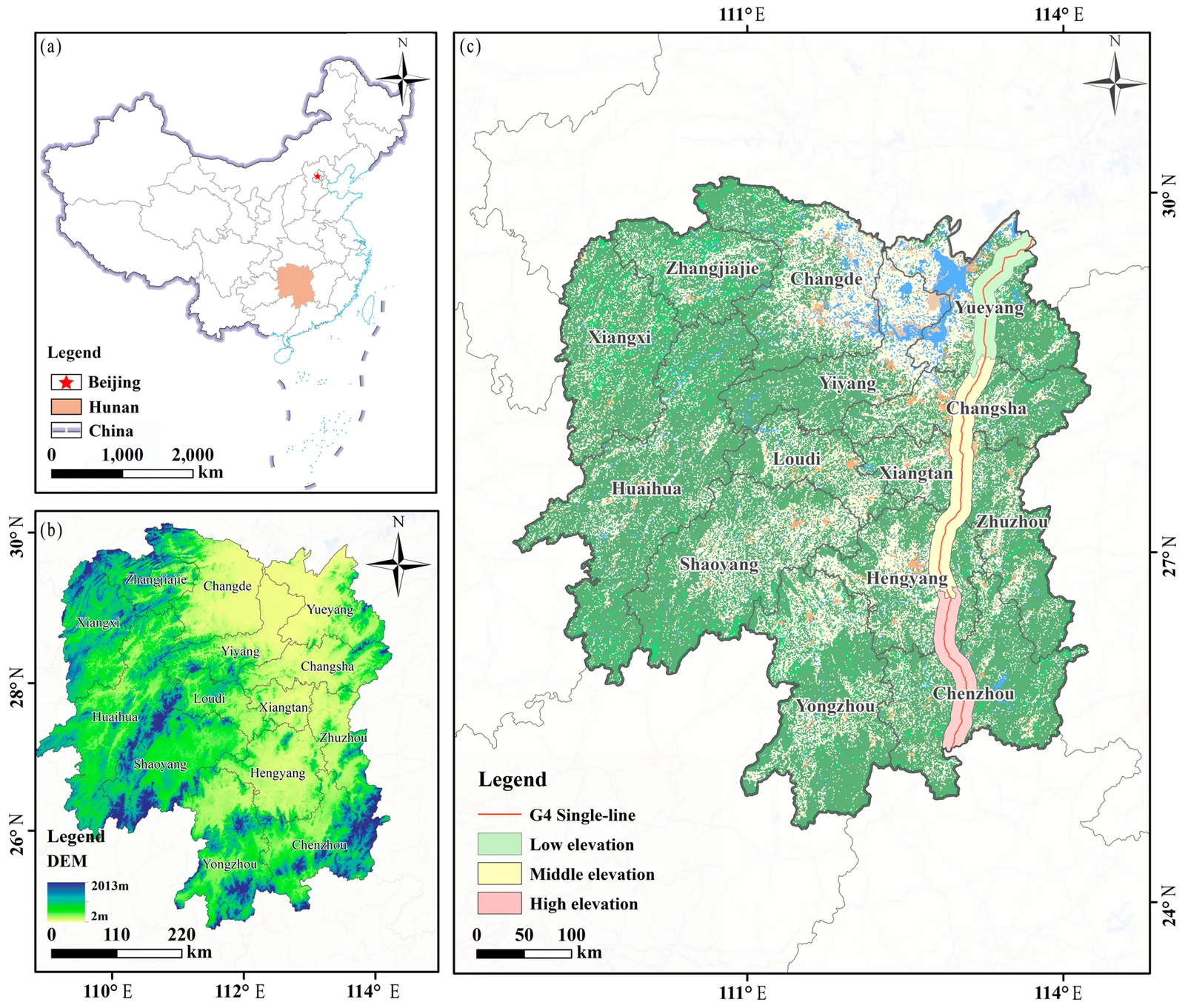
Study area and geographic highway sections along the G4 Beijing–Hong Kong–Macao Expressway in Hunan Province, China. (**a**) Location of Hunan Province within China; (**b**) topographic characteristics of Hunan Province derived from the digital elevation model (DEM); and (**c**) geographic distribution of the G4 Expressway and the LOW, MIDDLE, and HIGH highway sections, together with major cities. No copyright permission is required for the base map, which is sourced from the Standard Map Service System of China, with Map Approval Number GS(2019)1671.

The highway corridor was divided into low-, middle-, and high-elevation settings based jointly on regional topographic characteristics and the spatial continuity of administrative sections along the expressway, rather than on fixed elevation thresholds. The low-elevation setting was located mainly in the Yueyang section of northern Hunan, the middle-elevation setting mainly in the Changsha–Zhuzhou–Xiangtan–Hengyang corridor of central Hunan, and the high-elevation setting mainly in the Chenzhou section of southern Hunan. The LOW, MIDDLE, and HIGH sections extended approximately 134.20, 241.46, and 156.16 km along the expressway, respectively, with a combined length of approximately 531.82 km. Elevation was characterized using the Shuttle Radar Topography Mission (SRTM) DEM (USGS/SRTMGL1_003) at a spatial resolution of approximately 30 m. Within the highway buffer areas, mean elevations were 75.37, 100.95, and 263.68 m for the low-, middle-, and high-elevation settings, respectively. Although the low- and middle-elevation distributions partially overlapped, the middle setting had a somewhat higher overall elevation, whereas the high-elevation setting was clearly distinct. Therefore, the three groups were treated as contrasting elevation settings rather than as mutually exclusive elevation classes defined by fixed thresholds (Figure 1).

### 2.2. Landscape and Climate Data

#### 2.2.1. Cumulative-Buffer Landscape Data

Annual land-cover data for Hunan Province from 2000 to 2020 were obtained from the China Land Cover Dataset (CLCD) developed by Wuhan University (http://irsip.whu.edu.cn/resv2/resources_v2.php, accessed on 25 September 2025). CLCD is a Landsat-derived annual land-cover dataset with a spatial resolution of 30 m. It was generated using a random forest classification framework on the Google Earth Engine platform (GEE; Google LLC, Mountain View, CA, USA; accessed in August 2026), followed by spatio–temporal postprocessing to improve temporal consistency [36]. The dataset was downloaded from the official CLCD data portal of Wuhan University and land-cover classes followed the CLCD classification system.

Fourteen cumulative buffer widths were generated around the highway at 0.05, 0.10, 0.15, 0.20, 0.25, 0.50, 0.75, 1.00, 1.50, 2.00, 3.00, 5.00, 7.50, and 10.00 km. Each cumulative buffer represented the entire landscape area included between the highway and the specified outer boundary. The buffers were spatially nested, with each larger buffer incorporating all smaller cumulative extents. For example, the 1.00 km buffer contained the complete area from the highway to 1.00 km. The same cumulative-buffer framework was applied to the low-, middle-, and high-elevation settings. The resulting dataset contained 294 year–buffer combinations per elevation setting (21 years × 14 buffer widths).

This multiscale design was developed to characterize how landscape structure changed as progressively larger cumulative spatial extents were incorporated around the highway corridor, rather than to treat individual buffer widths as independent spatial observations [5,6,37]. Because cumulative buffers are nested by construction, responses across buffer widths were interpreted as scale-dependent changes in landscape structure rather than as independent distance-specific effects.

#### 2.2.2. Landscape Metrics

Seven landscape metrics were calculated: patch density (PD), aggregation index (AI), COHESION, CONTAG, largest patch index (LPI), Shannon’s Diversity Index (SHDI), and Landscape Shape Index (LSI). Because landscape metrics may contain substantial information redundancy [12,13,14], the main analyses focused on LSI, SHDI, and LPI. The cumulative-buffer profiles of the remaining four metrics are provided in Figure A3.

LSI was used to represent landscape shape and configuration complexity, SHDI to characterize compositional heterogeneity, and LPI to describe the relative dominance of the largest patch. Thus, these metrics represent complementary dimensions of landscape structure rather than alternative measures of a single fragmentation process [12,13].

For each year and elevation setting, each metric was calculated separately for the complete landscape within each cumulative buffer.

#### 2.2.3. Climate Variables

Annual climate variables for 2000–2020 were extracted for each highway section using Google Earth Engine (GEE). Mean temperature (MeanTemp) and precipitation (Precip) were obtained from ERA5-Land Monthly Aggregated (ECMWF/ERA5_LAND/MONTHLY_AGGR; approximately 11.1 km resolution) [38]. Annual values were calculated by averaging monthly mean temperatures and summing monthly precipitation totals, with units converted to °C and mm, respectively. Potential evapotranspiration (PET), actual evapotranspiration (AET), and climatic water deficit (Deficit) were obtained from TerraClimate (IDAHO_EPSCOR/TERRACLIMATE; approximately 4.6 km resolution) [39]. Annual totals were calculated by summing monthly values and applying a scale factor of 0.1.

SPEI12 was obtained from the December SPEI_12_month values of SPEIbase v2.10 (CSIC/SPEI/2_10; 0.5° resolution) [40]. December was selected because the 12-month accumulation period covers the corresponding calendar year, consistent with the annual landscape data.

Annual climate values were spatially averaged within each highway-section polygon using the GEE mean reducer, with reduction scales of 10,000 m for ERA5-Land, 4000 m for TerraClimate, and 5000 m for SPEIbase. These are computational sampling scales rather than the native spatial resolutions of the datasets. Each variable therefore had one annual value per highway section.

MeanTemp and SPEI12 were used to examine associations with the predefined annual landscape-profile contrasts.

### 2.3. Statistical Analyses

#### 2.3.1. Nonlinear Cumulative-Buffer Response Analysis

Generalized additive models (GAMs) were fitted separately for each landscape metric (LSI, SHDI, and LPI) and highway section (LOW, MIDDLE, and HIGH) to describe nonlinear relationships between landscape structure and cumulative buffer width. GAMs allow flexible estimation of nonlinear responses without imposing a fixed functional form [41,42]. The model was expressed as follows:

(1) $$Y_{t,w}=\beta_{0}+f\left(w\right)+\mu_{t}+\varepsilon_{t,w}$$

where *Y_t_*_,*w*_ is the landscape metric observed in year t at cumulative buffer width w (km); *β*_0_ is the intercept; *f*(*w*) is the penalized smooth function of buffer width; *μ_t_* is the year-specific random intercept; and *ε_t_*_,*w*_ is the residual error.

The models were fitted using the mgcv package in R 4.6.1, with a primary smooth basis dimension of k = 10 and smoothing parameters estimated by restricted maximum likelihood (REML) [41]. Each of the nine section–metric models used 21 annual profiles (2000–2020), comprising 14 nested cumulative buffer widths per year (294 observations). The year-specific random intercept accounted for shared annual variation among widths within the same year. To examine sensitivity to the choice of basis dimension, models were refitted with k = 8, 12, and 13, and their fitted curves, derivatives, turning points, and diagnostics were compared.

Because cumulative buffers were spatially nested, measurements at different widths were not independent. The GAMs were therefore interpreted as descriptive models of cumulative landscape-scale patterns, with temporal dependence and robustness examined separately in Section 2.3.2 and Section 2.3.3.

#### 2.3.2. Identification of Landscape-Scale Turning Points

The first derivatives of the fitted GAM smooths were examined to identify changes in landscape-metric response direction across cumulative buffer widths [33]. Derivatives were estimated numerically by finite differences:

(2) $$f^{\prime }\left(w\right)\approx \frac{f\left(w\right.\;+\;\left.h\right)\;-\;f\left(w\right.\;-\;\left.h\right)}{2h}$$

where *f*′(*w*) represents the rate of change in the fitted landscape metric with respect to cumulative buffer width w, and h denotes a small numerical increment in buffer width (km). The smooth function *f*(*w*) was defined in Equation (1).

Negative and positive derivatives indicated decreasing and increasing fitted values, respectively. Sign changes from negative to positive identified candidate local minima, whereas positive-to-negative changes indicated local maxima. To exclude short-lived derivative fluctuations, candidate turning points were required to show consistent derivative signs over at least 0.1 km on both sides of the transition. Their reproducibility was assessed using temporally blocked resampling of complete annual buffer profiles (Section 2.3.3). Turning-point locations were also compared across GAM basis dimensions (k = 8, 10, 12, and 13) to assess sensitivity to smoothing specification separately from temporal resampling.

#### 2.3.3. Temporal Dependence and Robustness Assessment

Temporal dependence was assessed using annual means of the GAM residuals across cumulative buffer widths. Linear trends, Kendall’s rank correlations, and lag-1 autocorrelations were calculated to characterize temporal trends and persistence [33]. Model adequacy was examined using normal Q–Q plots, residual-versus-fitted plots, and smooth basis-dimension diagnostics.

The stability of the fitted cumulative-buffer profiles was assessed using a moving-block bootstrap that resampled complete annual profiles [43]. Consecutive blocks of 2, 3, and 4 years were sampled with replacement, keeping all 14 cumulative widths together within each sampled year. For each block length, 200 bootstrap samples were generated, and the corresponding GAMs were refitted. Fitted curves, first derivatives, and turning-point locations were compared across block lengths. Turning-point resampling was also performed under k = 10 and k = 13 to separate sensitivity to annual sampling from sensitivity to the GAM basis dimension.

A bootstrap turning point was matched to the original estimate only when its type (minimum or maximum) agreed and its location was within ±0.5 km. Percentile ranges were calculated from successfully matched turning points, while pointwise 2.5th and 97.5th percentiles of the fitted curves were used to describe variation across resamples. Because the annual series exhibited temporal trends and residual dependence, these percentile ranges were treated as descriptive sensitivity intervals, not formally calibrated confidence intervals. The resampling procedure preserved the within-year cumulative profiles but did not eliminate the dependence associated with overlapping buffers or repeated annual observations.

As an additional sensitivity analysis, the nine GAMs were refitted as generalized additive mixed models (GAMMs) with a first-order autoregressive [AR(1)] residual correlation structure across the 14 ordered cumulative buffer widths within each year. Fitted profiles, initial LSI minima, and residual correlations were compared with the original GAM results to assess sensitivity to within-year buffer-width dependence.

### 2.4. Land-Cover Composition and Patch-Configuration Context

#### 2.4.1. Land-Cover Composition of Newly Included Annuli

To examine the land-cover composition added as cumulative highway buffers widened, successive annuli were defined as the areas between adjacent cumulative buffer boundaries. This approach builds on road-buffer land-cover analysis [9] and recognizes the scale dependence of landscape measurements [10]. The proportion of each land-cover class within an annulus was calculated as follows:

$$P_{i,\left(a\right.,\left.b\right]}=100\times \frac{{\mathrm{CA}}_{i}\left(b\right)-{\mathrm{CA}}_{i}\left(a\right)}{A\left(b\right)-A\left(a\right)}$$

where *P_i_*_,(*a*),(*b*]_ is the percentage of land-cover class i in the annulus between adjacent buffer widths a and b; *CA_i_*(*w*) is the cumulative area of class i; and *A*(*w*) is the total cumulative landscape area at width w. All areas were measured in hectares (ha).

Annular land-cover proportions were calculated separately for each highway section and year and averaged across the 21 years (2000–2020). Because differences in cumulative class areas describe land-cover composition but do not identify patch arrangement, the annular analysis was restricted to class proportions.

#### 2.4.2. Class-Level Largest-Patch Dominance

Class-level Largest Patch Index (LPI) and Percentage of Landscape (PLAND) were used to characterize the largest-patch dominance and relative abundance of individual land-cover classes within cumulative highway buffers [44]. To distinguish the proportion of the entire landscape occupied by a class’s largest patch from its proportion within that class, the within-class largest-patch share was calculated as follows:

$$D_{i}\left(w\right)=\frac{{\mathrm{LPI}}_{i}\left(w\right)}{{\mathrm{PLAND}}_{i}\left(w\right)}$$

where *D_i_*(*w*) represents the proportion of land-cover class i occupied by its largest patch at cumulative buffer width w; *LPI_i_*(*w*) is the percentage of total landscape area occupied by the largest patch of class i; and *PLAND_i_*(*w*) is the percentage of total landscape area occupied by class i. The ratio was calculated only when *PLAND_i_*(*w*) > 0.

Annual values of PLAND, class-level LPI, and *D_i_*(*w*) were compared between adjacent cumulative buffer widths, particularly around the initial LSI turning regions, to assess whether changes in land-cover abundance coincided with changes in largest-patch dominance. These comparisons examined whether changes in class abundance were accompanied by changes in largest-patch dominance, without interpreting the latter as evidence of improved functional connectivity.

### 2.5. Interannual Climate Associations with Landscape-Scale Responses

#### 2.5.1. Annual Landscape-Profile Contrasts and Climate Variables

To assess associations between annual climate variability and cumulative landscape-scale patterns, two annual profile contrasts were calculated for LSI, SHDI, and LPI within each highway section, motivated by the documented scale dependence of landscape metrics [10,21]. The small-minus-large contrast was defined as the mean metric value at cumulative widths ≤ 1 km minus that at widths ≥ 5 km. The early-minus-middle contrast was defined as the mean at widths ≤ 250 m minus that at widths from 500 m to 3 km. Both contrasts were calculated from observed landscape-metric values as follows:

$$C_{t}=\frac{1}{n_{1}}\sum_{w\in W_{1}}Y_{t,w}-\frac{1}{n_{2}}\sum_{w\in W_{2}}Y_{t,w}$$

where *C_t_* is the annual landscape-profile contrast; *W*_1_ and *W*_2_ are the two predefined sets of cumulative buffer widths being compared; and *n*_1_ and *n*_2_ are the numbers of widths in the respective sets. The observed landscape metric *Y_t_*_,*w*_ was defined in Equation (1).

Annual mean temperature (MeanTemp) and the 12-month Standardized Precipitation-Evapotranspiration Index (SPEI12) were used to examine climate associations with these contrasts [31]. For each highway section, both climate variables had one annual value shared across all 14 nested cumulative widths. Consequently, each section–metric–contrast analysis comprised 21 annual observations (2000–2020), rather than 21 × 14 independent climate observations.

#### 2.5.2. Statistical Models and Temporal Dependence

Associations between annual landscape-profile contrasts and climate variability were evaluated using three model specifications. Model M1 used ordinary least squares (OLS) regression with annual mean temperature (MeanTemp) and SPEI12 as simultaneous predictors. Model M2 added a linear year term to M1, while M3 retained the same predictors as M2 but used generalized least squares (GLS) with a first-order autoregressive [AR(1)] residual correlation structure [45]. The general model was expressed as follows:

$$C_{t}=\alpha +\beta_{T}T_{t}+\beta_{S}S_{t}+\gamma \tau_{t}+\varepsilon_{t}$$

where *C_t_* is the annual landscape-profile contrast defined in Equation (6); α is the intercept; *T_t_* and *S_t_* are standardized annual mean temperature and SPEI12, respectively; *β_T_* and *β_S_* are their regression coefficients; γ is the coefficient of the linear year trend; *τ_t_* = (t − 2010)/10 is the centered and scaled calendar year; and *ε_t_* is the residual error.

Climate variables were standardized by their standard deviations across the 21 annual observations within each highway section. Models were fitted separately for each highway section, landscape metric, and annual profile contrast. The AR(1) correlation parameter in M3 was estimated iteratively to account for residual dependence between consecutive years. Differences in coefficient estimates and statistical support across M1–M3 were used to assess the sensitivity of climate associations to trend adjustment and temporal autocorrelation, rather than to infer causal climate effects.

#### 2.5.3. Multiple-Testing Correction and Sensitivity Assessment

The Benjamini–Hochberg procedure was used to control the false discovery rate (FDR) across climate-coefficient tests [46]. For each combination of annual profile contrast and model specification, *p*-values were adjusted within a family of 18 tests (three highway sections × three landscape metrics × two climate variables). FDR-adjusted *p*-values (q-values) below 0.05 were considered statistically significant.

The robustness of climate associations was evaluated by comparing coefficient signs, magnitudes, 95% confidence intervals, and FDR-adjusted significance across M1–M3. Associations that changed substantially with trend adjustment or AR(1) residual modeling were considered model-sensitive. Given the limited 21-year time series and the separate FDR correction for each contrast–model combination, these analyses were interpreted as exploratory rather than confirmatory evidence of climate-related effects.

### 2.6. Data Quality Checks and Sensitivity Analyses

Data quality checks covered the completeness of annual buffer-width records, consistency of cumulative landscape areas, and agreement between landscape-level and class-level metrics. To assess sensitivity to buffer-window selection, the same near-road windows (0.05–2 km and 0.05–3 km) were applied to all three highway sections, considering the known scale dependence of landscape metrics [10]. As a supplementary check on initial turning-point locations, piecewise cubic Hermite interpolation was applied to landscape-metric profiles using logarithmically transformed buffer width. Climate-related window comparisons were conducted using year-adjusted regression with heteroskedasticity- and autocorrelation-consistent (HAC) standard errors [47]. These analyses examined sensitivity to the choice of analytical window and were not considered independent confirmation of the primary GAM or GLS-AR(1) results.

Temporal robustness and climate-model sensitivity were examined separately in Section 2.3.3, Section 2.5.2 and Section 2.5.3. Because landscape observations were aggregated into three highway sections rather than independently sampled road segments, spatial autocorrelation along the corridor could not be estimated directly. The sensitivity analyses were therefore used to evaluate the dependence of findings on analytical choices, not to establish causal highway or elevation effects.

## 3. Results

### 3.1. Nonlinear Landscape Patterns Across Cumulative Buffer Widths

**Figure 2:**
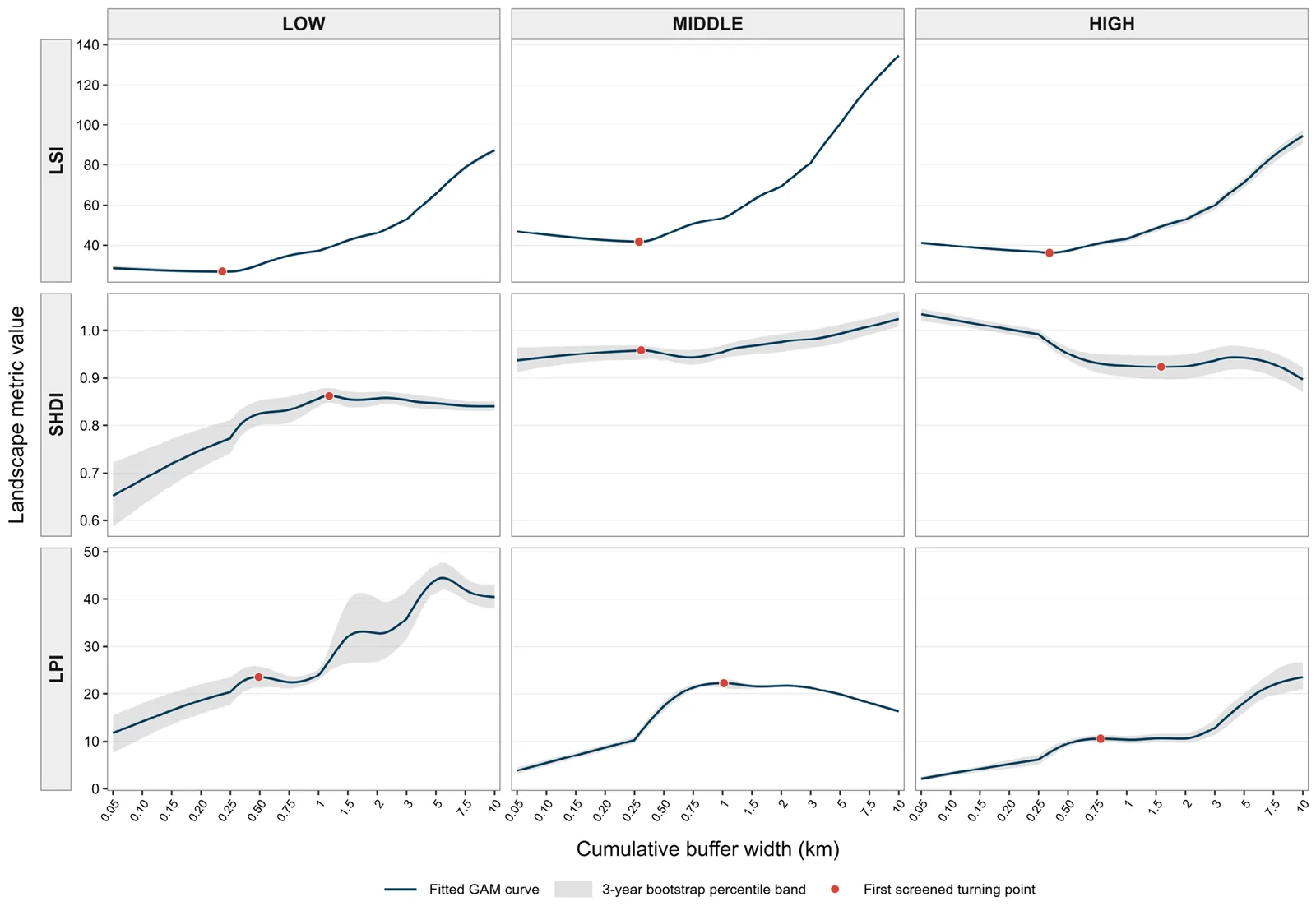
Nonlinear cumulative-buffer profiles of LSI, SHDI, and LPI across the LOW, MIDDLE, and HIGH highway sections. Blue lines represent fitted GAM curves, gray ribbons show descriptive pointwise 2.5th–97.5th percentile ranges from 3-year moving-block bootstrap resampling under the primary k = 10 specification, and red points indicate the first turning points meeting the screening criteria.

**Figure A1:**
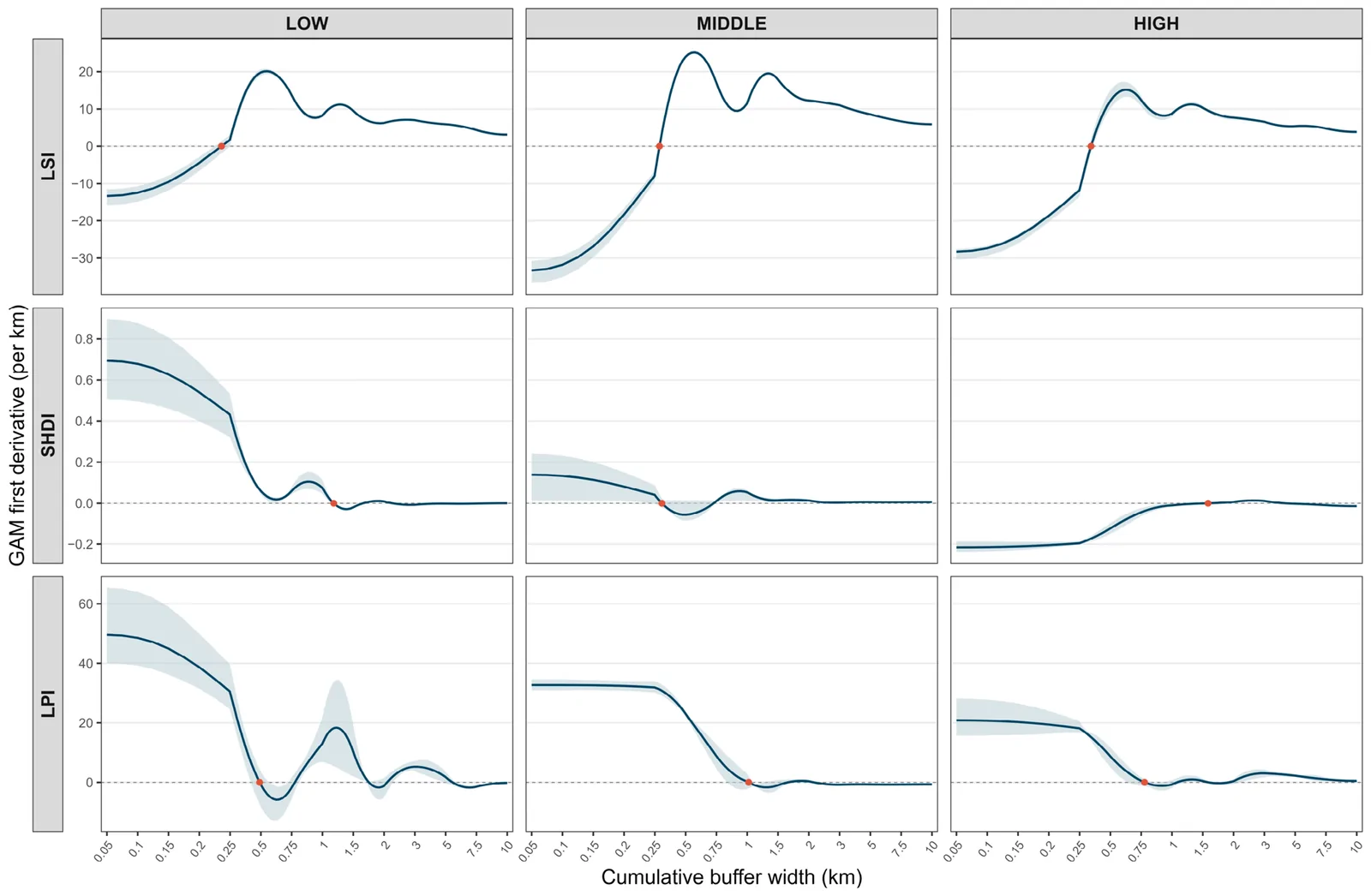
First-derivative profiles of the fitted GAM smooth functions for LSI, SHDI, and LPI across cumulative buffer widths in the LOW, MIDDLE, and HIGH highway sections. Solid lines represent estimated first derivatives, shaded bands indicate approximate 95% confidence intervals, and horizontal dashed lines indicate zero derivative values. Red points mark selected turning points identified from derivative sign changes. Negative-to-positive and positive-to-negative transitions indicate local minima and maxima, respectively. The horizontal axis shows cumulative buffer width (km).

LSI, SHDI, and LPI exhibited distinct nonlinear patterns across cumulative buffer widths (Figure 2). Under the primary GAM specification (k = 10), LSI showed a broadly U-shaped fitted profile in all three highway sections, with initial local minima at 0.236, 0.288, and 0.343 km in the LOW, MIDDLE, and HIGH sections, respectively. SHDI displayed more variable patterns, with initial local maxima at 1.183 km (LOW) and 0.307 km (MIDDLE), whereas the HIGH section showed an initial local minimum at 1.585 km. LPI exhibited initial local maxima at 0.490, 1.023, and 0.777 km in the LOW, MIDDLE, and HIGH sections, respectively (Figure A1). Together, these patterns show that the shape and location of landscape-metric responses differed among metrics and highway sections as cumulative buffer widths increased. Sensitivity analyses across alternative basis dimensions (k = 8, 12, and 13) generally preserved the broad response patterns but shifted some estimated turning points (Table A1). For LSI, the initial minima under k = 13 occurred at 0.151, 0.206, and 0.312 km in the LOW, MIDDLE, and HIGH sections, respectively, confirming that their estimated locations were sensitive to the choice of basis dimension.

**Figure A2:**
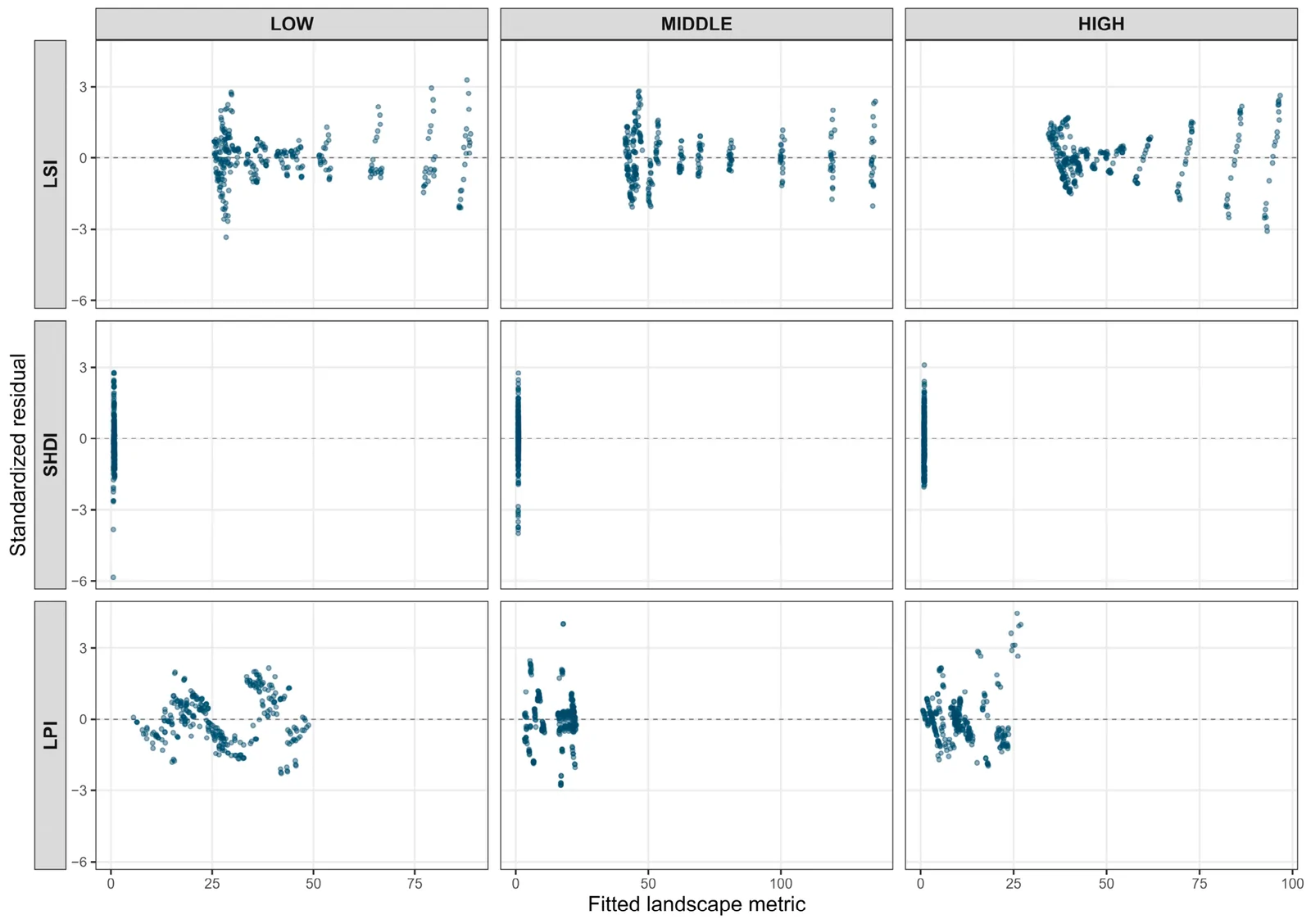
Standardized GAM residuals plotted against fitted values for LSI, SHDI, and LPI in the LOW, MIDDLE, and HIGH highway sections. Each point represents a model residual, and horizontal dashed lines indicate zero. The plots illustrate the distribution of residuals across fitted values and allow visual assessment of residual dispersion, heteroscedasticity, and remaining model structure.

**Figure A3:**
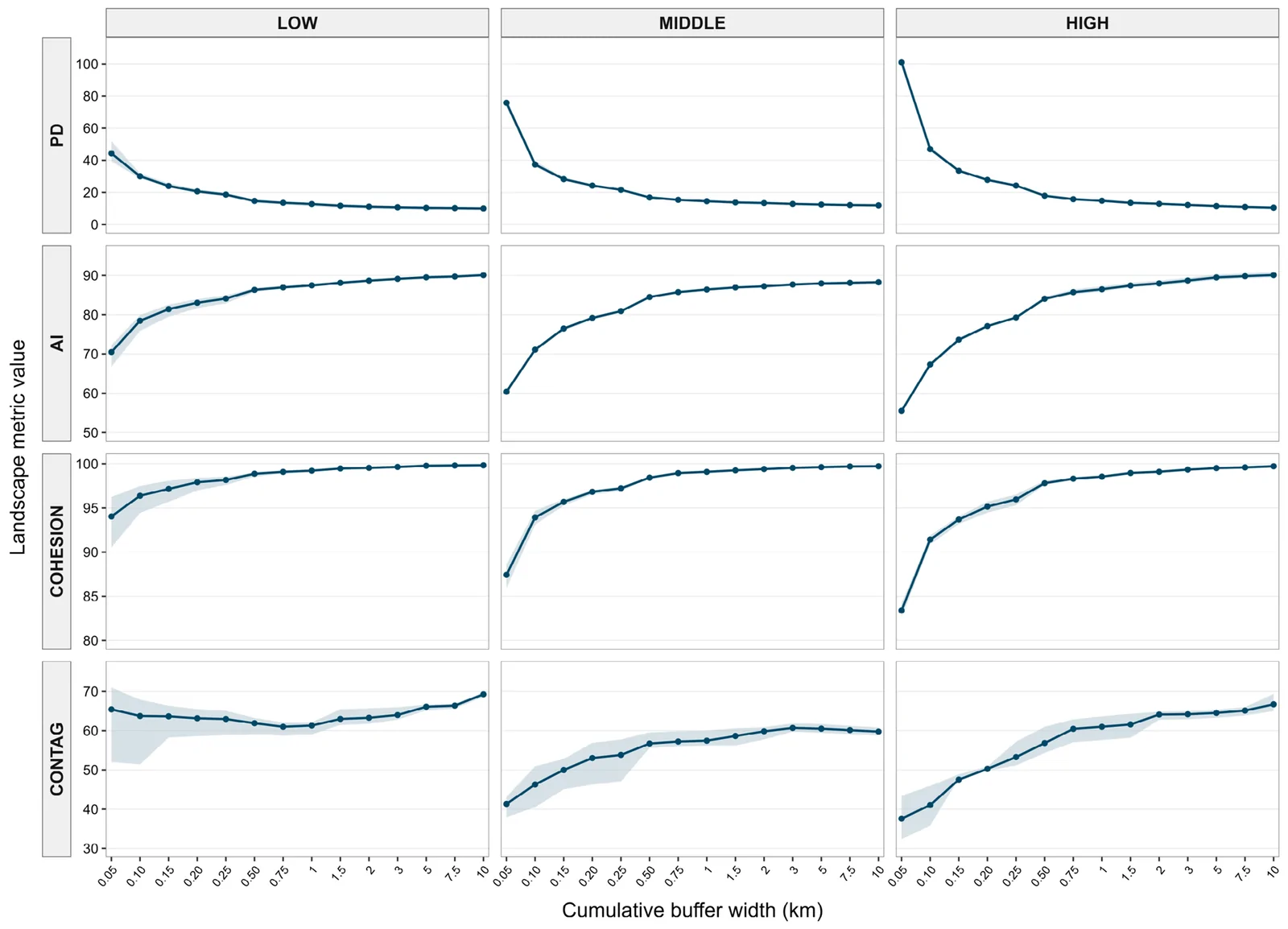
Cumulative-buffer profiles of four supplementary landscape metrics—patch density (PD), aggregation index (AI), patch cohesion index (COHESION), and contagion index (CONTAG)—across the LOW, MIDDLE, and HIGH highway sections during 2000–2020. Lines and points represent the mean metric values across the study period. Shaded ribbons indicate interannual variability. The horizontal axis represents cumulative buffer width (km).

Temporal diagnostics revealed substantial interannual dependence, with lag-1 autocorrelations of annual width-adjusted residuals ranging from 0.797 to 0.997 across the nine section–metric combinations (Table A2). GAM residual diagnostics indicated departures from normality. Residual-versus-fitted plots showed remaining residual structure (Figure A2), while basis-dimension checks suggested that some variation remained inadequately captured even at higher k values. Despite these diagnostic limitations, the initial LSI minima were detected within the predefined ±0.5 km matching tolerance in all 200 bootstrap resamples for each of the 2-, 3-, and 4-year block lengths under both k = 10 and k = 13 (Table 1 and Table A1). With 3-year blocks, the 2.5th–97.5th percentile ranges for the turning-point locations were 0.225–0.250 km (LOW), 0.283–0.294 km (MIDDLE), and 0.326–0.360 km (HIGH) for k = 10, compared with 0.143–0.156, 0.202–0.209, and 0.267–0.325 km, respectively, for k = 13 (Table A1). In contrast, the reproducibility of other SHDI and LPI turning points varied among highway sections and buffer-width ranges (Table A3). Overall, the initial LSI minima were consistently recovered across temporal block lengths, whereas their estimated positions shifted with the GAM basis dimension. Because turning points were matched using a tolerance of ±0.5 km, consistent recovery should not be taken as evidence of precise localization. The percentile ranges are descriptive measures of sensitivity, not formally calibrated confidence intervals.

The additional GAMM-AR(1) sensitivity analysis produced fitted cumulative-buffer profiles similar to those of the original GAMs across all nine section–metric combinations (Table A4). Relative root mean square differences between fitted profiles ranged from 0.06% to 3.14% of the corresponding baseline fitted ranges. Initial LSI minima were retained in all three sections, with estimated locations differing by approximately 0.01 km. The estimated within-year buffer-width AR(1) correlation parameters ranged from 0.104 to 0.980, indicating substantial residual dependence across ordered cumulative widths in several models. These findings support the descriptive stability of the main nonlinear patterns under the examined correlation structure.

### 3.2. Land-Cover Composition and Patch Dominance Across Cumulative Buffer Widths

Land-cover composition differed substantially among highway sections as cumulative buffer widths increased (Table A5). Within the newly included 250–500 m annulus, cropland accounted for 61.07%, 60.96%, and 42.02% of the area in the LOW, MIDDLE, and HIGH sections, respectively, averaged over 2000–2020 (Table 2). Forest accounted for 33.87%, 29.59%, and 52.79%, respectively. The HIGH section therefore contained a greater proportion of forest and a smaller proportion of cropland in this annulus than the LOW and MIDDLE sections.

**Figure 3:**
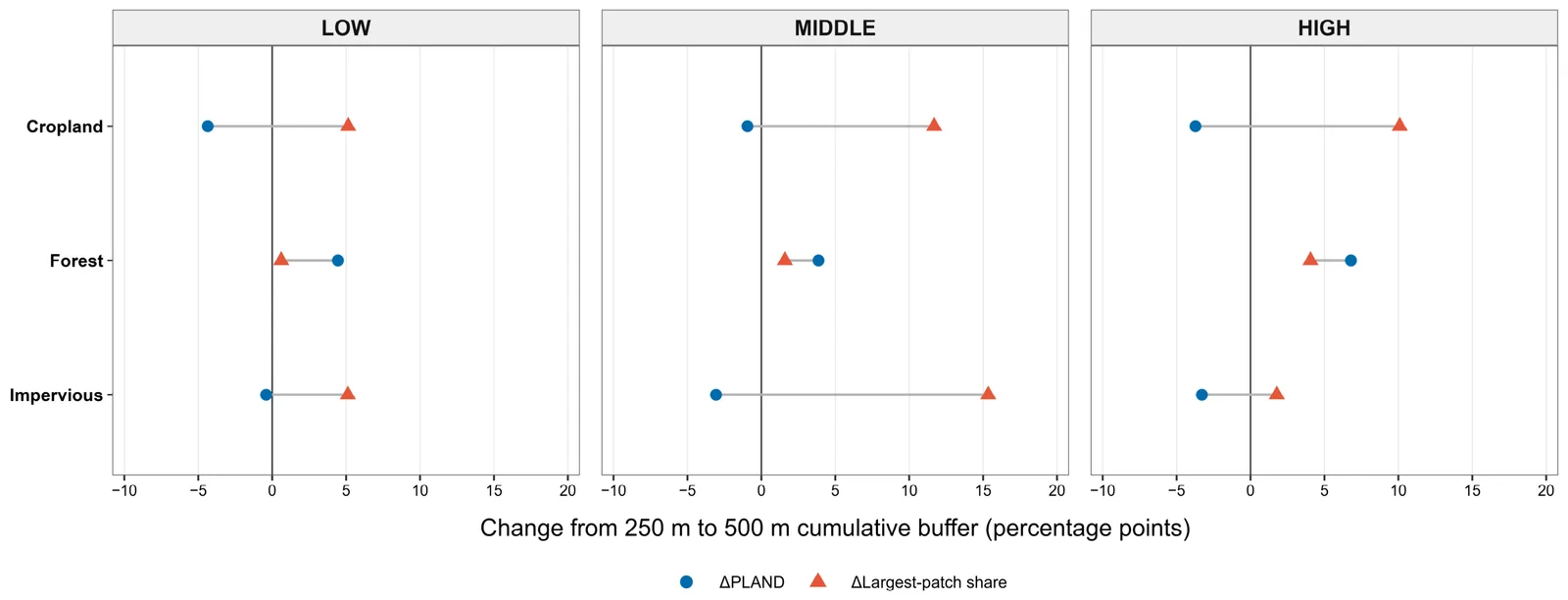
Changes in PLAND and within-class largest-patch share between the 250 m and 500 m cumulative highway buffers across the LOW, MIDDLE, and HIGH sections (2000–2020). Blue circles represent changes in PLAND, and red triangles represent changes in within-class largest-patch share. Horizontal gray lines connect the two values for each land-cover class. Values are means of annual differences (500 m minus 250 m) over the 21-year period and are expressed in percentage points. The vertical line at zero indicates no change.

Changes in class abundance and largest-patch dominance were not always aligned (Figure 3; Table 2 and Table A6). Between the 250 m and 500 m cumulative buffers, cropland PLAND decreased by 4.36, 0.94, and 3.72 percentage points in the LOW, MIDDLE, and HIGH sections, respectively, whereas class-level cropland LPI increased by 2.07, 7.08, and 4.14 percentage points. The within-class largest-patch share of cropland also increased by 5.14, 11.69, and 10.10 percentage points, respectively, with positive changes observed in all 21 years in each section. Thus, the proportion of the cumulative landscape occupied by cropland declined even as its largest patch occupied a greater share of both the landscape and the cropland class itself. Impervious surfaces showed the same opposing trends in PLAND and class-level LPI in the LOW and MIDDLE sections. In HIGH, both measures declined slightly, although the within-class largest-patch share increased (Table A6). Forest showed increases in both PLAND and class-level LPI across all three sections.

### 3.3. Interannual Climate Associations and Model Sensitivity

Annual landscape-profile contrasts showed little evidence of statistically significant climate associations after FDR correction (Table 3 and Table A7). For the small-minus-large contrast, none of the 18 climate-coefficient tests was FDR-significant under M1, M2, or M3. For the early-minus-middle contrast, the only FDR-significant association was between MeanTemp and SHDI in the LOW section under M2 (β = 0.0104, q = 0.0198). The corresponding association was no longer significant under M3 (β = 0.0047, q = 0.1402), indicating that its statistical support depended on how temporal autocorrelation was handled.

Climate-coefficient estimates also varied across model specifications. Among the 18 associations tested for each profile contrast, coefficient signs were consistent across all three models for only 7 small-minus-large associations and 10 early-minus-middle associations. The mean absolute estimated temporal AR(1) correlation coefficients were 0.721 and 0.687 for the two contrasts, respectively. Although several individual 95% confidence intervals excluded zero, none of the 36 climate associations was FDR-significant under M3. Overall, the observed climate associations depended on the temporal model specification, with little consistent statistical support after adjustment for temporal trends, residual autocorrelation, and multiple testing.

### 3.4. Sensitivity to Analytical Windows

Sensitivity analyses using the same 0.05–2 km and 0.05–3 km buffer windows across all three highway sections showed consistent initial LSI turning-point locations (Table 4 and Table A8). Using PCHIP interpolation on logarithmic buffer width, the first LSI minima occurred at 0.150, 0.200, and 0.250 km in the LOW, MIDDLE, and HIGH sections, respectively, and their estimated locations were unchanged between the two windows. The corresponding minima were detected in 20 of the 21 annual profiles for LOW and in all 21 annual profiles for both MIDDLE and HIGH. In contrast, the detection of SHDI and LPI turning points varied across metrics and sections. Thus, among the three metrics, the initial LSI minima showed the most consistent results across the two common analytical windows.

Exploratory climate-window comparisons yielded similar patterns across the two windows. In each window, four of the 18 climate-coefficient tests passed BH-FDR correction: LOW–LSI–MeanTemp, LOW–SHDI–MeanTemp, LOW–SHDI–SPEI12, and MIDDLE–SHDI–SPEI12 (Table A9). However, these analyses used window-specific annual contrasts and year-adjusted regressions with HAC standard errors rather than the GLS-AR(1) specification used in the primary analysis. Thus, the results indicate consistency across the two analytical windows but do not independently confirm the primary climate associations reported in Section 3.3.

## 4. Discussion

The findings reveal three complementary aspects of landscape variation along the Jinggang’ao Expressway: nonlinear responses to cumulative spatial extent, differences in land-cover composition and dominant-patch structure despite similar landscape-scale patterns, and the sensitivity of annual climate associations to temporal model specification. Together, these results demonstrate the value of distinguishing observed landscape patterns from their structural characteristics and the statistical robustness of their climate associations.

### 4.1. Nonlinear Scale Dependence and Geographic Variation in Landscape Structure

The nonlinear cumulative-scale patterns observed along the Jinggang’ao Expressway were consistent with H1, indicating that fitted landscape profiles varied among metrics and geographic sections. Previous research has established that road-related ecological effects vary with distance and environmental context. The spatial extent of these effects differs among ecological processes and species [5,6], while highway-associated changes in landscape structure may vary across land-cover settings [9]. However, our analysis addresses a different question: how landscape metrics vary as progressively larger, spatially nested areas surrounding an existing highway are included. The resulting profiles therefore characterize landscape structure across cumulative analytical extents, rather than identifying ecological road-effect distances.

The broadly U-shaped LSI profiles illustrate the scale dependence of measured landscape shape complexity. Roads can contribute to habitat fragmentation, edge creation, and changes in patch geometry [2,3], while highway development has also been associated with changes in landscape structure [48]. However, cumulative-buffer analysis cannot isolate these processes. As buffer width increases, additional land-cover patches and patch boundaries are incorporated, altering the spatial composition and configuration of the landscape being analyzed. Because LSI standardizes total edge length against the minimum possible edge length for a given landscape area, its values depend on the spatial arrangement and geometry of patches within the analytical extent. The initial decline and subsequent increase in LSI may therefore reflect changes in the spatial configuration of the landscape included at successive buffer widths, rather than variation in the magnitude of highway-related ecological effects. Landscape metrics are sensitive to spatial scale [13], and changes in analytical extent or spatial delineation can affect their values [49]. These findings emphasize the importance of interpreting nonlinear cumulative-scale responses within their analytical context.

The turning-point analyses highlight the distinction between the repeated detection of a nonlinear feature and the precision of its estimated location. The initial local minima of the fitted LSI profiles were repeatedly detected under moving-block bootstrap resampling, although their estimated locations varied with GAM basis dimension (Table A1). Supplementary common-window analyses also detected initial LSI minima, but the estimated locations differed between estimation methods. Thus, the initial local minima were reproducible as features of the cumulative profiles, whereas their precise locations were sensitive to model specification and estimation method. Landscape metrics have recognized limitations in representing landscape heterogeneity and ecological processes [17], and their values may depend on spatial delineation [49]. Our results further indicate that turning-point estimates derived from fitted landscape profiles require similar caution. These minima should therefore be interpreted as approximate structural turning regions rather than ecological thresholds or boundaries of highway-related effects.

The distinct responses of SHDI and LPI further demonstrate that cumulative landscape structure cannot be characterized adequately by a single metric. LSI quantifies landscape shape complexity, SHDI measures land-cover diversity by accounting for class richness and proportional abundance, and LPI represents the proportion of the landscape occupied by its largest patch. These metrics describe different aspects of landscape composition and spatial configuration and may respond differently as agricultural, forested, and developed areas are progressively included. Habitat amount and spatial configuration are distinct landscape properties [18], while road–landscape relationships may vary with land-cover characteristics and geographic setting [27,50]. The LOW, MIDDLE, and HIGH sections exhibited different metric-specific responses across their respective geographic contexts. However, differences in terrain, land use, and human activity prevent these patterns from being attributed to elevation alone. Overall, the findings indicate that cumulative landscape-scale responses are nonlinear, metric-specific, and geographically differentiated.

### 4.2. Land-Cover Composition and Dominant-Patch Structure Across Cumulative Highway Buffers

The findings support H2 by showing that broadly similar initial cumulative LSI profiles can occur in highway sections with contrasting land-cover composition and class-specific largest-patch dominance. This indicates that similar landscape-level responses do not necessarily reflect comparable landscape composition or spatial configuration. Previous studies have shown that highway-associated landscape patterns vary with surrounding land cover and geographic context [9,50]. Our analysis builds on this work by examining the composition of newly included annuli alongside class-level patch structure within cumulative buffers. In the 250–500 m annulus, cropland was the dominant land-cover class in the LOW and MIDDLE sections, whereas forest predominated in the HIGH section (Table 2). Despite these differences, all three sections showed broadly similar initial LSI profiles, with an initial decline followed by an increase. Thus, similar cumulative-scale responses can occur across contrasting land-cover settings without necessarily reflecting the same underlying landscape processes.

One of the clearest findings was the contrasting response of cropland proportional abundance and largest-patch dominance as cumulative buffer width increased from 250 to 500 m. Cropland PLAND decreased in all three sections, while class-level LPI increased (Table 2; Figure 3). These contrasting trends reflect differences between class abundance and dominant-patch structure. While PLAND describes the relative abundance of cropland, class-level LPI and within-class largest-patch share capture complementary aspects of the dominance of its largest patch. The increase in within-class largest-patch share was consistent with the simultaneous decline in PLAND and increase in class-level LPI. This relationship was observed in all 21 annual comparisons within each section. These changes reflect differences between nested analytical extents, not temporal changes in cropland area or patch configuration. Cropland therefore occupied a smaller proportion of the enlarged landscape, even as its largest patch accounted for a greater share of both the total landscape and the cropland area included.

This divergence illustrates why landscape composition and spatial configuration need to be considered separately when interpreting cumulative-scale responses. Previous research has shown that these properties can follow different temporal trajectories [23] and that landscape metrics respond to changes in analytical extent [22]. Our analysis addresses the latter by comparing different cumulative buffer widths within each annual land-cover dataset. Importantly, this does not demonstrate that cropland patches expanded or merged over time. Such changes in LPI may result from the inclusion of additional portions of existing patches, a change in which patch is the largest within the buffer, or differences in how patches intersect buffer boundaries. An increase in within-class largest-patch share also does not necessarily indicate reduced habitat fragmentation, improved functional connectivity, or higher habitat quality. These ecological interpretations require evidence beyond landscape metrics alone [14].

The main contribution of H2 is to show how complementary measures of landscape composition and patch structure provide information that aggregate cumulative profiles cannot capture. By combining annular land-cover composition with cumulative class-level metrics, the analysis distinguishes changes in class abundance from differences in dominant-patch structure across buffer widths. These measures are particularly useful when similar landscape-level LSI profiles occur in highway sections with contrasting land-cover mosaics. Landscape-level LPI and class-level LPI must also be distinguished because the largest patch in the overall landscape may belong to a different class from the largest patch within a particular land-cover class. Although these analyses reveal differences in landscape composition and configuration, they do not identify which landscape components produced the fitted LSI turning points. Overall, the findings show that similar cumulative-scale landscape profiles can conceal substantial differences in class abundance and dominant-patch structure without implying equivalent ecological processes or functional outcomes.

### 4.3. Annual Climate Associations and Implications for Highway Landscape Monitoring

The results for H3 highlight the sensitivity of annual climate–landscape associations to analytical specification. Although one climate coefficient remained significant after FDR correction under the year-adjusted M2 model, none of the 36 tests across the two predefined contrasts passed FDR correction under the AR(1)-adjusted M3 models (Table 3). This difference underscores the importance of accounting for temporal dependence, which can affect statistical inference in ecological time-series analyses [33]. The exploratory window analyses identified several FDR-significant associations (Table A9), but these were based on different landscape contrasts and HAC-adjusted regressions rather than the primary GLS-AR(1) specification. Taken together, these results indicate that the observed climate associations were sensitive to model specification, with statistical support varying across models rather than providing robust evidence of climate effects. Moreover, nonlinear cumulative-scale landscape patterns under H1 should not be interpreted as evidence of corresponding annual climate responses.

Interpreting these findings requires a distinction between climate-sensitive ecological processes and landscape structure derived from classified land-cover data. Climatic variability can affect vegetation productivity, drought stress, and ecosystem recovery [28,29,30], but these responses may not produce detectable changes in land-cover classes or patch boundaries. Studies of road-effect zones have also examined ecosystem services and related ecological indicators, including habitat quality, water yield, and soil erosion [51]. By contrast, the metrics used here quantify the composition and spatial configuration of land-cover mosaics rather than ecosystem functioning. Vegetation condition may change within a mapped patch without altering its land-cover class or spatial boundaries. Conversely, land management and urban development may change landscape composition or configuration without corresponding directly to annual climatic anomalies. The ecological interpretation of landscape metrics therefore depends on the structural attributes they measure and their relationships with ecological processes [14]. The lack of FDR-significant annual climate associations under M3 should not be taken as evidence that ecosystem functioning is unaffected by climatic variability.

Regional climatic variability must also be distinguished from local road-edge microclimatic conditions. Forest edges and linear canopy openings can create microclimatic gradients in air temperature, relative humidity, and other environmental variables [52]. Roadside vegetation responses may also differ with local forest-edge conditions [53]. These local gradients are not necessarily represented by climate indicators measured or aggregated over larger areas. In this study, MeanTemp and SPEI12 describe annual climatic conditions at the highway-section level. SPEI12 characterizes climatic water-balance anomalies [31], whereas road-edge microclimates are also influenced by canopy structure, edge exposure, and local surface properties. Section-level climate indicators and site-level microclimatic measurements therefore provide complementary information at different spatial scales and should not be treated as interchangeable measures of environmental conditions along highways.

These distinctions are relevant to the design of highway ecological monitoring programs. Cumulative landscape metrics describe landscape composition and spatial configuration across analytical extents, while biological surveys and measurements of traffic exposure and local environmental conditions provide complementary information on ecological responses to roads. Biological response data can help identify road-effect distances [16], and traffic-volume data can inform assessments of traffic-related ecological exposure [54]. Monitoring programs should therefore distinguish landscape structure, environmental exposure, and biological responses rather than treating them as equivalent indicators. Combining land-cover and patch metrics with climate observations, traffic measurements, and biological monitoring would provide a more comprehensive assessment of ecological conditions along highway corridors.

### 4.4. Limitations and Future Directions

Several limitations should be considered when interpreting these findings. First, the 14 cumulative buffer widths were spatially nested and therefore did not provide independent spatial samples. Although moving-block bootstrap resampling and sensitivity analyses helped assess the reproducibility of nonlinear cumulative-scale patterns, they did not account for spatial dependence among overlapping buffers or provide independent spatial replication across the three aggregated highway sections. GAM residual diagnostics also indicated substantial temporal autocorrelation, and the estimated locations of the initial LSI minima varied with model specification and estimation method. The identified turning regions should therefore be interpreted as descriptive features of cumulative landscape profiles rather than ecological thresholds or road-effect boundaries. Future studies using spatially replicated highway segments and nonoverlapping distance bands could provide a stronger basis for assessing spatial variation.

Second, the observational design did not allow highway-related effects to be separated from differences in surrounding land use, topography, and other geographic characteristics. Consequently, the contrasting landscape patterns among the LOW, MIDDLE, and HIGH sections cannot be attributed to elevation alone. The annular composition and class-level patch analyses also did not quantify the contributions of individual land-cover classes or patch configurations to the fitted LSI turning points. Furthermore, landscape structural metrics do not directly measure functional connectivity or biological responses. Matched reference landscapes, spatially explicit patch tracking, and field-based ecological observations would help address these limitations.

Finally, the analysis of annual climate associations was constrained by 21 temporally correlated observations per section, potential confounding by land-use trends, and section-level climate data that did not capture local road-edge microclimatic conditions. The gridded climate datasets were substantially coarser than the 30 m land-cover data, particularly SPEIbase at 0.5° resolution. Uncertainties in gridded climate estimates, including those associated with spatial interpolation and aggregation, may have obscured local climatic variability and influenced the estimated associations. The analyses also did not directly examine lagged climate responses or ecosystem functioning. Because the study examined only one highway corridor, the generalizability of these findings remains uncertain. Longer time series, independent highway replicates, and complementary environmental and biological measurements would strengthen future assessments.

## 5. Conclusions

This study examined landscape structure across 14 nested cumulative buffer widths in three geographically distinct sections of the Jinggang’ao Expressway during 2000–2020. LSI, SHDI, and LPI exhibited nonlinear, metric-specific, and geographically differentiated cumulative-scale patterns, consistent with H1. The initial LSI minima were consistently recovered across temporal block-bootstrap analyses, while supplementary common-window comparisons also showed stable turning-point locations. However, their precise estimates remained sensitive to GAM specification and estimation method. These findings distinguish reproducible landscape-scale patterns from precisely localized ecological thresholds or highway-effect boundaries.

The analysis further showed that similar initial LSI response forms can conceal substantial differences in land-cover composition and class-level patch dominance, supporting H2. Cropland predominated in the newly included 250–500 m annuli of the LOW and MIDDLE sections, whereas forest was more prominent in the HIGH section. Across all three sections, cropland PLAND declined as cumulative buffers expanded from 250 to 500 m, while class-level LPI and within-class largest-patch share increased. The latter increased in all 21 annual comparisons within each section. These findings demonstrate the value of combining cumulative landscape profiles, annular composition, and class-specific patch measures to reveal structural differences that aggregate metrics alone may obscure.

Annual climate associations with landscape-profile contrasts showed limited consistency across temporal model specifications. The only FDR-significant association under M2 was not retained under M3, where none of the 36 climate-coefficient tests remained significant after FDR correction. Exploratory common-window analyses yielded four BH-FDR-significant coefficients per window under a different annual contrast and HAC(2) error treatment (Table A9), emphasizing sensitivity to contrast definition and modeling choices rather than confirming the primary associations. Thus, H3 highlights the importance of temporal model specification when interpreting climate–landscape associations, rather than establishing systematic climatic effects. Together, these findings support a multidimensional approach to highway landscape monitoring that distinguishes cumulative-scale structure, underlying land-cover and patch characteristics, and the robustness of climate-related inference.

## Figures and Tables

**Table 1:** Initial turning points and temporal robustness of GAM-fitted cumulative-buffer response curves across highway sections.

Metric	Section	Initial Turning Type	GAM Turning Point (km)	3-Year Block Lower 2.5% (km)	3-Year Block Upper 97.5% (km)	Matched (2-Year Blocks)	Matched (3-Year Blocks)	Matched (4-Year Blocks)
LSI	LOW	minimum	0.236102	0.224869	0.250452	1	1	1
	MIDDLE	minimum	0.28819	0.283135	0.294061	1	1	1
	HIGH	minimum	0.342869	0.326212	0.359955	1	1	1
SHDI	LOW	maximum	1.182674	1.148881	1.191928	1	1	1
	MIDDLE	maximum	0.3072	0.248689	0.335314	0.8	0.835	0.84
	HIGH	minimum	1.585364	1.342813	1.704586	1	1	1
LPI	LOW	maximum	0.490143	0.418781	0.555858	1	0.99	0.985
	MIDDLE	maximum	1.023329	0.837758	1.089365	1	1	1
	HIGH	maximum	0.777413	0.644193	0.836706	1	0.995	1

Notes: Turning points were identified from the first derivatives of GAM-fitted curves. Percentile ranges represent the 2.5th–97.5th percentiles of matched turning-point locations obtained from 200 moving-block bootstrap resamples using 3-year blocks. Match percentages indicate the proportion of 200 resamples in which a corresponding turning point was identified within 0.5 km of the original estimate. These ranges describe sensitivity to temporal resampling and are not formally calibrated confidence intervals.

**Table 2:** Land-cover composition of the 250–500 m annulus and changes in cropland patch dominance between cumulative buffer widths of 250 and 500 m across highway sections (2000–2020).

Section	Cropland (%)	Forest (%)	Impervious (%)	ΔPLAND (pp)	ΔLPI (pp)	ΔLargest-Patch Share (pp)
LOW	61.07	33.87	3.76	−4.36	+2.07	+5.14
MIDDLE	60.96	29.59	7.88	−0.94	+7.08	+11.69
HIGH	42.02	52.79	4.03	−3.72	+4.14	+10.10

Notes: Class percentages are 21-year means of annual proportions in the newly included 250–500 m annulus. The Δ statistics are means of annual differences between the 500 m and 250 m cumulative buffers, and all refer to cropland. PLAND and class-level LPI are percentages of total landscape area; largest-patch share is the fraction of a class occupied by its largest patch. Changes in these percentage quantities are reported in percentage points (pp).

**Table 3:** False discovery rate-adjusted climate associations across model specifications and a representative LOW–SHDI association (2000–2020).

A. BH-FDR-Significant Climate-Coefficient Tests				
**Annual profile contrast**		**Model**	**BH-FDR significant tests (q < 0.05)**	
Small-minus-large		M1	0/18	
Small-minus-large		M2	0/18	
Small-minus-large		M3	0/18	
Early-minus-middle		M1	0/18	
Early-minus-middle		M2	1/18	
Early-minus-middle		M3	0/18	
B. LOW–SHDI association with annual mean temperature (early-minus-middle)				
**Model**	**β per 1 SD MeanTemp**	**95% CI**	***p***	**BH-FDR q**
M1	0.0241	[0.0013, 0.0470]	0.0397	0.4790
M2	0.0104	[0.0048, 0.0160]	0.0011	0.0198
M3	0.0047	[0.0009, 0.0085]	0.0175	0.1402

Notes. M1, ordinary least squares (OLS) with MeanTemp and SPEI12; M2, OLS additionally including a linear year trend; M3, generalized least squares with the same predictors as M2 and an AR(1) error structure. Each model used 21 annual observations for each section–metric–contrast combination. BH-FDR adjustment was performed separately across 18 tests (3 sections × 3 metrics × 2 climate variables) within each model and contrast. β represents the landscape-profile contrast change associated with a one-standard-deviation increase in MeanTemp. Confidence intervals and nominal *p*-values are model-based; a CI excluding zero does not imply BH-FDR significance.

**Table 4:** Sensitivity of initial LSI minima to common 0.05–2 km and 0.05–3 km cumulative-buffer windows.

Highway Section	Turning Point 2 km	Turning Point 3 km (km)	Absolute Shift (km)	Annual Detections 2 km	Annual Detections 3 km
LOW	0.150	0.150	0.000	20/21	20/21
MIDDLE	0.200	0.200	0.000	21/21	21/21
HIGH	0.250	0.250	0.000	21/21	21/21

Notes: Turning points are descriptive estimates obtained using piecewise cubic Hermite interpolation (PCHIP), not the GAM-derived turning points reported in Section 3.1. Annual detections indicate the number of years (out of 21) in which a minimum was identified in the predefined search interval.

## Data Availability

The data supporting the findings of this study are available from the corresponding author upon reasonable request.

## Appendix A

**Table A1:** Sensitivity of first screened GAM turning points to smooth basis dimension and temporal block resampling.

Metric	Section	k = 8 (km)	k = 10 (km)	k = 12 (km)	k = 13 (km)	Δ(13–10) (km)	k = 10, 3-y P2.5–P97.5 (km)	k = 13, 3-y P2.5–P97.5 (km)	k = 10 Match %	k = 13 Match %
LSI	LOW	—	0.236	0.170	0.151	−0.085	0.226–0.248	0.143–0.156	100.0–100.0	100.0–100.0
LSI	MIDDLE	0.242	0.288	0.202	0.206	−0.082	0.283–0.293	0.202–0.209	100.0–100.0	100.0–100.0
LSI	HIGH	0.438	0.343	0.312	0.312	−0.031	0.327–0.358	0.267–0.325	100.0–100.0	100.0–100.0
SHDI	LOW	0.907	1.183	1.736	1.735	+0.552	1.153–1.191	1.432–1.895	100.0–100.0	98.0–99.0
SHDI	MIDDLE	—	0.307	0.177	0.163	−0.144	0.242–0.339	0.141–0.181	82.0–84.0	82.5–85.5
SHDI	HIGH	1.068	1.585	1.489	1.484	−0.101	1.403–1.718	1.272–1.745	100.0–100.0	100.0–100.0
LPI	LOW	5.661	0.490	0.518	0.521	+0.030	0.424–0.555	0.398–0.621	99.5–100.0	84.0–86.0
LPI	MIDDLE	1.043	1.024	0.886	0.886	−0.137	0.846–1.090	0.856–0.892	100.0–100.0	100.0–100.0
LPI	HIGH	0.906	0.778	0.782	0.782	+0.004	0.613–0.820	0.653–0.828	99.5–100.0	100.0–100.0

Notes. Values are the first screened turning-point locations (km) identified using the same derivative-sign persistence rule (0.1 km on either side). A dash indicates that no turning point met the screening criterion. Δ(13–10) is the k = 13 location minus the k = 10 location. The 3-y P2.5–P97.5 columns give the 2.5th–97.5th percentiles of successfully matched turning points from the 3-year moving-block bootstrap (200 resamples per model). Match % gives the minimum–maximum matching percentages across 2-, 3-, and 4-year blocks, each with 200 successful fits; matches required the same turning-point type within ±0.5 km of the baseline estimate. These are conditional descriptive sensitivity intervals, not calibrated confidence intervals. The turning-point estimates depend on the chosen basis dimension and should not be interpreted as precise road-effect thresholds.

**Table A2:** GAM fit summaries and annual temporal-dependence diagnostics.

Section	Metric	R^2^	Deviance Explained	Turning Features	Annual Trend Slope	Kendall τ	Lag-1 Raw	Lag-1 Detrended
LOW	LSI	0.998	0.998	1	0.0416	0.171	0.894	0.886
	SHDI	0.868	0.881	4	0.0066	0.886	0.977	0.803
	LPI	0.869	0.881	5	−0.5473	−0.638	0.948	0.793
MIDDLE	LSI	0.999	0.999	1	−0.0483	−0.390	0.797	0.654
	SHDI	0.936	0.942	2	0.0041	0.933	0.984	0.716
	LPI	0.972	0.974	3	−0.0715	−0.762	0.900	0.482
HIGH	LSI	0.990	0.991	1	0.2542	0.733	0.973	0.800
	SHDI	0.957	0.961	2	0.0041	1.000	0.997	0.887
	LPI	0.921	0.928	4	−0.2331	−0.886	0.907	0.606

Notes: Values were extracted from corrected Step 1B outputs. Width-adjusted annual summaries comprise 21 years per section–metric combination. Lag-1 coefficients describe temporal dependence and do not address spatial autocorrelation. Fit summaries are descriptive and should not be interpreted as independent predictive validation.

**Table A3:** Full derivative-screened GAM turning features and moving-block resampling support.

Section	Metric	Turn No.	Type	GAM Width (km)	Block Years	Match (%)	Matched 2.5–97.5% (km)
HIGH	LPI	1	maximum	0.777	2	100.0	0.650–0.823
HIGH	LPI	1	maximum	0.777	3	99.5	0.644–0.837
HIGH	LPI	1	maximum	0.777	4	100.0	0.563–0.815
HIGH	LPI	2	minimum	1.081	2	100.0	0.973–1.204
HIGH	LPI	2	minimum	1.081	3	99.5	0.910–1.202
HIGH	LPI	2	minimum	1.081	4	100.0	0.826–1.208
HIGH	LPI	3	maximum	1.593	2	100.0	1.497–1.683
HIGH	LPI	3	maximum	1.593	3	99.5	1.499–1.673
HIGH	LPI	3	maximum	1.593	4	100.0	1.499–1.674
HIGH	LPI	4	minimum	1.918	2	100.0	1.816–2.083
HIGH	LPI	4	minimum	1.918	3	99.5	1.797–2.191
HIGH	LPI	4	minimum	1.918	4	99.0	1.786–2.226
HIGH	LSI	1	minimum	0.343	2	100.0	0.328–0.358
HIGH	LSI	1	minimum	0.343	3	100.0	0.326–0.360
HIGH	LSI	1	minimum	0.343	4	100.0	0.322–0.358
HIGH	SHDI	1	minimum	1.585	2	100.0	1.334–1.696
HIGH	SHDI	1	minimum	1.585	3	100.0	1.343–1.705
HIGH	SHDI	1	minimum	1.585	4	100.0	1.373–1.705
HIGH	SHDI	2	maximum	4.363	2	100.0	4.202–4.579
HIGH	SHDI	2	maximum	4.363	3	100.0	4.150–4.603
HIGH	SHDI	2	maximum	4.363	4	100.0	4.163–4.615
LOW	LPI	1	maximum	0.490	2	100.0	0.429–0.561
LOW	LPI	1	maximum	0.490	3	99.0	0.419–0.556
LOW	LPI	1	maximum	0.490	4	98.5	0.416–0.541
LOW	LPI	2	minimum	0.776	2	100.0	0.734–0.804
LOW	LPI	2	minimum	0.776	3	99.0	0.726–0.808
LOW	LPI	2	minimum	0.776	4	98.5	0.718–0.811
LOW	LPI	3	maximum	1.754	2	95.5	1.726–1.798
LOW	LPI	3	maximum	1.754	3	90.0	1.719–1.802
LOW	LPI	3	maximum	1.754	4	93.0	1.715–1.795
LOW	LPI	4	minimum	2.099	2	95.0	1.927–2.254
LOW	LPI	4	minimum	2.099	3	91.0	1.916–2.295
LOW	LPI	4	minimum	2.099	4	93.0	1.905–2.296
LOW	LPI	5	maximum	5.561	2	98.5	5.134–5.825
LOW	LPI	5	maximum	5.561	3	95.0	5.167–5.889
LOW	LPI	5	maximum	5.561	4	94.0	5.142–5.900
LOW	LSI	1	minimum	0.236	2	100.0	0.227–0.247
LOW	LSI	1	minimum	0.236	3	100.0	0.225–0.250
LOW	LSI	1	minimum	0.236	4	100.0	0.223–0.249
LOW	SHDI	1	maximum	1.183	2	100.0	1.162–1.192
LOW	SHDI	1	maximum	1.183	3	100.0	1.149–1.192
LOW	SHDI	1	maximum	1.183	4	100.0	1.152–1.191
LOW	SHDI	2	minimum	1.660	2	100.0	1.634–1.708
LOW	SHDI	2	minimum	1.660	3	100.0	1.633–1.725
LOW	SHDI	2	minimum	1.660	4	100.0	1.632–1.713
LOW	SHDI	3	maximum	2.276	2	100.0	2.191–2.335
LOW	SHDI	3	maximum	2.276	3	100.0	2.160–2.336
LOW	SHDI	3	maximum	2.276	4	100.0	2.176–2.341
LOW	SHDI	4	minimum	9.004	2	85.0	8.569–9.427
LOW	SHDI	4	minimum	9.004	3	79.0	8.554–9.411
LOW	SHDI	4	minimum	9.004	4	71.0	8.545–9.386
MIDDLE	LPI	1	maximum	1.023	2	100.0	0.875–1.085
MIDDLE	LPI	1	maximum	1.023	3	100.0	0.838–1.089
MIDDLE	LPI	1	maximum	1.023	4	100.0	0.825–1.093
MIDDLE	LPI	2	minimum	1.659	2	97.5	1.471–1.787
MIDDLE	LPI	2	minimum	1.659	3	99.0	1.274–1.754
MIDDLE	LPI	2	minimum	1.659	4	99.0	1.241–1.725
MIDDLE	LPI	3	maximum	2.163	2	97.0	2.005–2.234
MIDDLE	LPI	3	maximum	2.163	3	99.0	1.962–2.238
MIDDLE	LPI	3	maximum	2.163	4	99.0	2.010–2.230
MIDDLE	LSI	1	minimum	0.288	2	100.0	0.283–0.293
MIDDLE	LSI	1	minimum	0.288	3	100.0	0.283–0.294
MIDDLE	LSI	1	minimum	0.288	4	100.0	0.281–0.293
MIDDLE	SHDI	1	maximum	0.307	2	80.0	0.265–0.329
MIDDLE	SHDI	1	maximum	0.307	3	83.5	0.249–0.335
MIDDLE	SHDI	1	maximum	0.307	4	84.0	0.231–0.342
MIDDLE	SHDI	2	minimum	0.727	2	80.0	0.697–0.738
MIDDLE	SHDI	2	minimum	0.727	3	84.5	0.694–0.741
MIDDLE	SHDI	2	minimum	0.727	4	84.0	0.683–0.739

Notes: Each block length used 200 resamples; turning-point matching tolerance was ±0.5 km. Percentile intervals summarize matched resampled locations and are not formally calibrated confidence intervals. A missing matched interval denotes insufficient or absent matching support.

**Table A4:** Sensitivity of cumulative-buffer landscape profiles to within-year buffer-width dependence: comparison of GAM and GAMM-AR(1) fits.

Section	Metric	AR(1) Parameter	Relative RMSE (%)	GAM LSI Minimum (km)	GAMM LSI Minimum (km)
LOW	LSI	0.839	0.17	0.239	0.249
	SHDI	0.974	3.14	—	—
	LPI	0.715	0.69	—	—
MIDDLE	LSI	0.509	0.09	0.289	0.299
	SHDI	0.808	1.37	—	—
	LPI	0.104	0.06	—	—
HIGH	LSI	0.977	0.62	0.339	0.349
	SHDI	0.980	0.57	—	—
	LPI	0.761	0.30	—	—

Notes: The AR(1) parameter describes the working residual correlation between adjacent ordered cumulative buffer widths within a year, not the temporal AR(1) used for annual climate associations. Relative RMSE (%) is the root mean square difference between GAM and GAMM fitted profiles expressed as a percentage of the range of the baseline GAM fitted profile. Initial LSI minima were estimated using the common prediction grid for this sensitivity analysis and may differ slightly from derivative-based estimates in the main text. A dash indicates that the LSI-minimum comparison does not apply to SHDI or LPI. These results assess sensitivity to the specified correlation structure and do not establish spatial independence along the highway.

**Table A5:** Mean land-cover composition of newly included annuli across three highway sections during 2000–2020.

Section	Annulus (m)	Cropland (%)	Forest (%)	Grassland (%)	Shrub (%)	Impervious (%)	Water (%)	Barren (%)
LOW	50–100	71.26	23.64	0.04	0.00	4.47	0.60	0.00
	100–150	67.76	27.10	0.04	0.00	4.33	0.77	0.00
	150–200	65.76	29.42	0.06	0.00	3.98	0.78	0.00
	200–250	64.30	31.39	0.03	0.00	3.57	0.71	0.00
	250–500	61.07	33.87	0.06	0.00	3.76	1.24	0.00
	500–750	56.18	37.85	0.05	0.00	4.70	1.23	0.00
	750–1000	55.14	40.13	0.03	0.00	3.56	1.14	0.00
	1000–1500	52.67	43.37	0.08	0.00	2.76	1.13	0.00
	1500–2000	53.47	42.62	0.03	0.00	2.55	1.33	0.00
	2000–3000	52.54	43.87	0.04	0.00	2.29	1.26	0.00
	3000–5000	51.29	45.50	0.05	0.00	1.97	1.18	0.00
	5000–7500	47.02	50.22	0.04	0.00	1.30	1.39	0.04
	7500–10,000	44.67	52.21	0.03	0.00	1.39	1.71	0.00
MIDDLE	50–100	68.70	20.11	0.00	0.00	9.80	1.39	0.00
	100–150	65.15	25.02	0.01	0.00	8.21	1.60	0.00
	150–200	62.79	27.36	0.01	0.00	8.38	1.46	0.00
	200–250	61.93	28.79	0.01	0.00	7.95	1.32	0.00
	250–500	60.96	29.59	0.01	0.00	7.88	1.56	0.00
	500–750	59.18	30.67	0.01	0.00	8.30	1.84	0.00
	750–1000	58.96	30.86	0.01	0.00	7.69	2.48	0.00
	1000–1500	57.85	30.75	0.01	0.00	7.70	3.69	0.00
	1500–2000	58.32	30.18	0.01	0.00	7.53	3.95	0.00
	2000–3000	59.27	28.93	0.01	0.00	7.71	4.08	0.00
	3000–5000	56.89	31.15	0.02	0.00	8.01	3.92	0.00
	5000–7500	56.88	29.38	0.02	0.00	9.09	4.64	0.00
	7500–10,000	54.34	30.87	0.01	0.00	9.27	5.50	0.00
HIGH	50–100	54.88	37.86	0.04	0.00	6.59	0.62	0.00
	100–150	49.34	44.35	0.03	0.00	5.60	0.67	0.00
	150–200	46.67	47.21	0.03	0.00	5.21	0.88	0.00
	200–250	45.46	48.62	0.02	0.00	4.76	1.14	0.00
	250–500	42.02	52.79	0.02	0.00	4.03	1.15	0.00
	500–750	39.54	55.17	0.07	0.00	4.08	1.14	0.01
	750–1000	38.87	54.98	0.16	0.00	4.79	1.19	0.00
	1000–1500	38.59	54.69	0.08	0.00	5.15	1.49	0.00
	1500–2000	39.29	53.69	0.06	0.00	5.27	1.68	0.00
	2000–3000	37.16	54.29	0.04	0.00	6.34	2.16	0.00
	3000–5000	40.34	51.50	0.03	0.00	6.68	1.45	0.00
	5000–7500	33.53	59.75	0.07	0.02	5.07	1.56	0.00
	7500–10,000	30.53	66.14	0.17	0.11	2.17	0.88	0.00

Notes: For each section and annulus, percentages were calculated annually and then averaged over 21 years (2000–2020). Absent class–year combinations were treated as zero area; consequently, the seven class means sum to 100% per row before rounding. Values may differ from 100% by up to 0.02 percentage points after rounding. Each annulus is the area added between adjacent cumulative buffer widths.

**Table A6:** Changes in class-level land-cover abundance and largest-patch dominance between cumulative buffer widths of 250 and 500 m (2000–2020).

Section	Land-Cover Class	ΔPLAND (pp)	ΔLPI (pp)	ΔLargest-Patch Share (pp)	Years With Increased Share (n/21)
LOW	Cropland	−4.36	+2.07	+5.14	21/21
	Forest	+4.45	+0.46	+0.61	17/21
	Impervious	−0.41	+0.16	+5.12	21/21
MIDDLE	Cropland	−0.94	+7.08	+11.69	21/21
	Forest	+3.87	+0.52	+1.59	21/21
	Impervious	−3.06	+0.97	+15.35	21/21
HIGH	Cropland	−3.72	+4.14	+10.10	21/21
	Forest	+6.79	+2.47	+4.06	21/21
	Impervious	−3.29	−0.04	+1.78	19/21

Notes: Δ denotes the average within-year value at 500 m minus that at 250 m (21 annual comparisons per row). Largest-patch share equals class-level LPI/PLAND and is a proportion between 0 and 1; its difference was multiplied by 100 for display in percentage points. Annual increases count years with a positive difference in this share. These statistics describe cumulative buffers, not patch configuration within the newly included annulus.

**Table A7:** Climate coefficient estimates for all annual landscape-profile contrasts and temporal model specifications (2000–2020). The 108 coefficient tests: two contrasts × three highway sections × three landscape metrics × two climate variables × three models.

Contrast	Section	Metric	Climate	Model	n	β (Per SD)	95% CI	*p*	BH q	AR(1) ρ
Small–large	LOW	LSI	MeanTemp	M1	21	0.3040	[−0.4431, 1.0511]	0.4038	0.6804	—
Small–large	LOW	LSI	MeanTemp	M2	21	−0.0164	[−0.5972, 0.5644]	0.9532	0.9532	—
Small–large	LOW	LSI	MeanTemp	M3	21	−0.0158	[−0.2363, 0.2046]	0.8808	0.8808	0.839
Small–large	LOW	LSI	SPEI12	M1	21	−0.0590	[−0.8061, 0.6881]	0.8701	0.8701	—
Small–large	LOW	LSI	SPEI12	M2	21	−0.3412	[−0.9164, 0.2340]	0.2277	0.6186	—
Small–large	LOW	LSI	SPEI12	M3	21	0.0244	[−0.1677, 0.2166]	0.7909	0.8808	0.839
Small–large	LOW	SHDI	MeanTemp	M1	21	0.0237	[0.0023, 0.0451]	0.0321	0.3877	—
Small–large	LOW	SHDI	MeanTemp	M2	21	0.0113	[0.0033, 0.0193]	0.0085	0.1526	—
Small–large	LOW	SHDI	MeanTemp	M3	21	0.0048	[0.0004, 0.0091]	0.0331	0.5954	0.869
Small–large	LOW	SHDI	SPEI12	M1	21	0.0189	[−0.0026, 0.0403]	0.0812	0.3877	—
Small–large	LOW	SHDI	SPEI12	M2	21	0.0079	[−0.0000, 0.0158]	0.0502	0.3012	—
Small–large	LOW	SHDI	SPEI12	M3	21	0.0016	[−0.0022, 0.0053]	0.3916	0.8808	0.869
Small–large	LOW	LPI	MeanTemp	M1	21	−0.9699	[−3.6739, 1.7341]	0.4608	0.6913	—
Small–large	LOW	LPI	MeanTemp	M2	21	0.4595	[−1.0504, 1.9694]	0.5294	0.6806	—
Small–large	LOW	LPI	MeanTemp	M3	21	0.8612	[−0.3354, 2.0577]	0.1466	0.8797	0.503
Small–large	LOW	LPI	SPEI12	M1	21	−0.5852	[−3.2892, 2.1188]	0.6548	0.7857	—
Small–large	LOW	LPI	SPEI12	M2	21	0.6739	[−0.8215, 2.1692]	0.3551	0.6806	—
Small–large	LOW	LPI	SPEI12	M3	21	0.4390	[−0.6101, 1.4882]	0.3882	0.8808	0.503
Small–large	MIDDLE	LSI	MeanTemp	M1	21	−0.0372	[−0.4345, 0.3601]	0.8463	0.8701	—
Small–large	MIDDLE	LSI	MeanTemp	M2	21	0.1141	[−0.2577, 0.4859]	0.5259	0.6806	—
Small–large	MIDDLE	LSI	MeanTemp	M3	21	−0.0608	[−0.3546, 0.2330]	0.6669	0.8808	0.719
Small–large	MIDDLE	LSI	SPEI12	M1	21	−0.2059	[−0.6033, 0.1914]	0.2906	0.5811	—
Small–large	MIDDLE	LSI	SPEI12	M2	21	−0.1140	[−0.4721, 0.2440]	0.5107	0.6806	—
Small–large	MIDDLE	LSI	SPEI12	M3	21	−0.0463	[−0.2490, 0.1563]	0.6345	0.8808	0.719
Small–large	MIDDLE	SHDI	MeanTemp	M1	21	0.0010	[−0.0021, 0.0040]	0.5129	0.7101	—
Small–large	MIDDLE	SHDI	MeanTemp	M2	21	0.0025	[0.0002, 0.0049]	0.0340	0.3012	—
Small–large	MIDDLE	SHDI	MeanTemp	M3	21	0.0011	[−0.0002, 0.0024]	0.0812	0.7312	0.915
Small–large	MIDDLE	SHDI	SPEI12	M1	21	0.0004	[−0.0026, 0.0035]	0.7735	0.8701	—
Small–large	MIDDLE	SHDI	SPEI12	M2	21	0.0014	[−0.0009, 0.0036]	0.2110	0.6186	—
Small–large	MIDDLE	SHDI	SPEI12	M3	21	0.0005	[−0.0004, 0.0014]	0.2199	0.8808	0.915
Small–large	MIDDLE	LPI	MeanTemp	M1	21	−0.2560	[−0.6855, 0.1734]	0.2264	0.5797	—
Small–large	MIDDLE	LPI	MeanTemp	M2	21	0.0262	[−0.1758, 0.2283]	0.7873	0.8858	—
Small–large	MIDDLE	LPI	MeanTemp	M3	21	0.0222	[−0.2065, 0.2508]	0.8398	0.8808	0.078
Small–large	MIDDLE	LPI	SPEI12	M1	21	−0.2390	[−0.6684, 0.1905]	0.2576	0.5797	—
Small–large	MIDDLE	LPI	SPEI12	M2	21	−0.0675	[−0.2621, 0.1271]	0.4740	0.6806	—
Small–large	MIDDLE	LPI	SPEI12	M3	21	−0.0594	[−0.2533, 0.1345]	0.5255	0.8808	0.078
Small–large	HIGH	LSI	MeanTemp	M1	21	−1.5455	[−3.4636, 0.3725]	0.1077	0.3877	—
Small–large	HIGH	LSI	MeanTemp	M2	21	0.2476	[−0.2026, 0.6977]	0.2620	0.6186	—
Small–large	HIGH	LSI	MeanTemp	M3	21	0.0507	[−0.2168, 0.3183]	0.6931	0.8808	0.945
Small–large	HIGH	LSI	SPEI12	M1	21	−1.1028	[−3.0208, 0.8153]	0.2427	0.5797	—
Small–large	HIGH	LSI	SPEI12	M2	21	0.0885	[−0.3380, 0.5151]	0.6670	0.8004	—
Small–large	HIGH	LSI	SPEI12	M3	21	0.0282	[−0.1534, 0.2098]	0.7464	0.8808	0.945
Small–large	HIGH	SHDI	MeanTemp	M1	21	−0.0072	[−0.0149, 0.0005]	0.0642	0.3877	—
Small–large	HIGH	SHDI	MeanTemp	M2	21	−0.0002	[−0.0028, 0.0024]	0.8691	0.9202	—
Small–large	HIGH	SHDI	MeanTemp	M3	21	−0.0002	[−0.0022, 0.0018]	0.8028	0.8808	0.795
Small–large	HIGH	SHDI	SPEI12	M1	21	−0.0031	[−0.0107, 0.0046]	0.4158	0.6804	—
Small–large	HIGH	SHDI	SPEI12	M2	21	0.0016	[−0.0009, 0.0041]	0.1906	0.6186	—
Small–large	HIGH	SHDI	SPEI12	M3	21	0.0006	[−0.0007, 0.0020]	0.3390	0.8808	0.795
Small–large	HIGH	LPI	MeanTemp	M1	21	1.3471	[−0.3131, 3.0073]	0.1055	0.3877	—
Small–large	HIGH	LPI	MeanTemp	M2	21	0.6142	[−1.0688, 2.2972]	0.4519	0.6806	—
Small–large	HIGH	LPI	MeanTemp	M3	21	−0.2312	[−1.1909, 0.7285]	0.6166	0.8808	0.822
Small–large	HIGH	LPI	SPEI12	M1	21	−0.3658	[−2.0260, 1.2944]	0.6489	0.7857	—
Small–large	HIGH	LPI	SPEI12	M2	21	−0.8528	[−2.4475, 0.7420]	0.2749	0.6186	—
Small–large	HIGH	LPI	SPEI12	M3	21	0.0646	[−0.5836, 0.7127]	0.8354	0.8808	0.822
Early–middle	LOW	LSI	MeanTemp	M1	21	0.3766	[−0.0890, 0.8422]	0.1064	0.4790	—
Early–middle	LOW	LSI	MeanTemp	M2	21	0.0929	[0.0120, 0.1738]	0.0269	0.1613	—
Early–middle	LOW	LSI	MeanTemp	M3	21	0.0830	[0.0128, 0.1532]	0.0234	0.1402	−0.334
Early–middle	LOW	LSI	SPEI12	M1	21	0.2578	[−0.2078, 0.7234]	0.2599	0.5197	—
Early–middle	LOW	LSI	SPEI12	M2	21	0.0079	[−0.0722, 0.0880]	0.8379	0.8872	—
Early–middle	LOW	LSI	SPEI12	M3	21	−0.0125	[−0.0848, 0.0598]	0.7189	0.9341	−0.334
Early–middle	LOW	SHDI	MeanTemp	M1	21	0.0241	[0.0013, 0.0470]	0.0397	0.4790	—
Early–middle	LOW	SHDI	MeanTemp	M2	21	0.0104	[0.0048, 0.0160]	0.0011	0.0198	—
Early–middle	LOW	SHDI	MeanTemp	M3	21	0.0047	[0.0009, 0.0085]	0.0175	0.1402	0.682
Early–middle	LOW	SHDI	SPEI12	M1	21	0.0186	[−0.0042, 0.0415]	0.1039	0.4790	—
Early–middle	LOW	SHDI	SPEI12	M2	21	0.0065	[0.0010, 0.0121]	0.0235	0.1613	—
Early–middle	LOW	SHDI	SPEI12	M3	21	0.0019	[−0.0014, 0.0052]	0.2386	0.7159	0.682
Early–middle	LOW	LPI	MeanTemp	M1	21	−0.5393	[−1.4391, 0.3606]	0.2241	0.5197	—
Early–middle	LOW	LPI	MeanTemp	M2	21	−0.4405	[−1.3973, 0.5164]	0.3451	0.6092	—
Early–middle	LOW	LPI	MeanTemp	M3	21	0.0105	[−0.8310, 0.8521]	0.9792	0.9792	0.450
Early–middle	LOW	LPI	SPEI12	M1	21	0.3508	[−0.5491, 1.2506]	0.4235	0.5536	—
Early–middle	LOW	LPI	SPEI12	M2	21	0.4378	[−0.5098, 1.3855]	0.3434	0.6092	—
Early–middle	LOW	LPI	SPEI12	M3	21	0.3175	[−0.4230, 1.0580]	0.3769	0.8479	0.450
Early–middle	MIDDLE	LSI	MeanTemp	M1	21	−0.0155	[−0.2317, 0.2006]	0.8817	0.9335	—
Early–middle	MIDDLE	LSI	MeanTemp	M2	21	−0.1284	[−0.2936, 0.0368]	0.1194	0.5374	—
Early–middle	MIDDLE	LSI	MeanTemp	M3	21	−0.0115	[−0.1200, 0.0971]	0.8253	0.9341	0.620
Early–middle	MIDDLE	LSI	SPEI12	M1	21	0.1273	[−0.0888, 0.3434]	0.2318	0.5197	—
Early–middle	MIDDLE	LSI	SPEI12	M2	21	0.0587	[−0.1004, 0.2179]	0.4468	0.6186	—
Early–middle	MIDDLE	LSI	SPEI12	M3	21	0.0105	[−0.0650, 0.0860]	0.7718	0.9341	0.620
Early–middle	MIDDLE	SHDI	MeanTemp	M1	21	0.0017	[−0.0031, 0.0066]	0.4613	0.5536	—
Early–middle	MIDDLE	SHDI	MeanTemp	M2	21	0.0016	[−0.0038, 0.0069]	0.5412	0.6959	—
Early–middle	MIDDLE	SHDI	MeanTemp	M3	21	0.0022	[0.0006, 0.0039]	0.0111	0.1402	0.852
Early–middle	MIDDLE	SHDI	SPEI12	M1	21	0.0037	[−0.0012, 0.0085]	0.1331	0.4791	—
Early–middle	MIDDLE	SHDI	SPEI12	M2	21	0.0036	[−0.0016, 0.0087]	0.1642	0.5911	—
Early–middle	MIDDLE	SHDI	SPEI12	M3	21	0.0009	[−0.0002, 0.0021]	0.1059	0.4766	0.852
Early–middle	MIDDLE	LPI	MeanTemp	M1	21	0.0035	[−0.4019, 0.4089]	0.9857	0.9857	—
Early–middle	MIDDLE	LPI	MeanTemp	M2	21	0.1763	[−0.1845, 0.5371]	0.3171	0.6092	—
Early–middle	MIDDLE	LPI	MeanTemp	M3	21	−0.1529	[−0.3820, 0.0763]	0.1764	0.6352	0.672
Early–middle	MIDDLE	LPI	SPEI12	M1	21	−0.1505	[−0.5558, 0.2549]	0.4456	0.5536	—
Early–middle	MIDDLE	LPI	SPEI12	M2	21	−0.0455	[−0.3930, 0.3019]	0.7855	0.8837	—
Early–middle	MIDDLE	LPI	SPEI12	M3	21	0.0113	[−0.1473, 0.1699]	0.8822	0.9341	0.672
Early–middle	HIGH	LSI	MeanTemp	M1	21	−0.6540	[−1.5945, 0.2866]	0.1613	0.4840	—
Early–middle	HIGH	LSI	MeanTemp	M2	21	0.1878	[−0.1880, 0.5637]	0.3065	0.6092	—
Early–middle	HIGH	LSI	MeanTemp	M3	21	0.0723	[−0.0875, 0.2322]	0.3516	0.8479	0.938
Early–middle	HIGH	LSI	SPEI12	M1	21	−0.4168	[−1.3573, 0.5238]	0.3642	0.5536	—
Early–middle	HIGH	LSI	SPEI12	M2	21	0.1425	[−0.2136, 0.4986]	0.4103	0.6154	—
Early–middle	HIGH	LSI	SPEI12	M3	21	0.0317	[−0.0768, 0.1401]	0.5447	0.9341	0.938
Early–middle	HIGH	SHDI	MeanTemp	M1	21	−0.0072	[−0.0149, 0.0006]	0.0675	0.4790	—
Early–middle	HIGH	SHDI	MeanTemp	M2	21	−0.0002	[−0.0029, 0.0026]	0.9095	0.9095	—
Early–middle	HIGH	SHDI	MeanTemp	M3	21	−0.0004	[−0.0023, 0.0015]	0.6950	0.9341	0.884
Early–middle	HIGH	SHDI	SPEI12	M1	21	−0.0030	[−0.0108, 0.0047]	0.4194	0.5536	—
Early–middle	HIGH	SHDI	SPEI12	M2	21	0.0016	[−0.0010, 0.0043]	0.2154	0.6092	—
Early–middle	HIGH	SHDI	SPEI12	M3	21	0.0004	[−0.0008, 0.0017]	0.4784	0.9341	0.884
Early–middle	HIGH	LPI	MeanTemp	M1	21	0.2267	[−0.2874, 0.7407]	0.3665	0.5536	—
Early–middle	HIGH	LPI	MeanTemp	M2	21	0.1220	[−0.4521, 0.6961]	0.6596	0.7915	—
Early–middle	HIGH	LPI	MeanTemp	M3	21	0.0330	[−0.3899, 0.4560]	0.8705	0.9341	0.751
Early–middle	HIGH	LPI	SPEI12	M1	21	−0.1668	[−0.6808, 0.3473]	0.5042	0.5672	—
Early–middle	HIGH	LPI	SPEI12	M2	21	−0.2363	[−0.7803, 0.3077]	0.3723	0.6092	—
Early–middle	HIGH	LPI	SPEI12	M3	21	0.0248	[−0.2608, 0.3104]	0.8562	0.9341	0.751

Notes: M1 = OLS with both climate variables; M2 = OLS with both climate variables and a linear year term; M3 = GLS with the same predictors and an AR(1) residual structure. β denotes the change in the unstandardized landscape-profile contrast per one within-section standard deviation of the climate predictor. Intervals are nominal 95% confidence intervals, not multiplicity-adjusted. BH-FDR adjustment was performed separately within each model and annual contrast over 18 tests (3 sections × 3 metrics × 2 climate variables). ρ is shown only for M3. Each model uses 21 annual observations per section.

**Table A8:** Complete PCHIP turning-point comparison across common 0.05–2 km and 0.05–3 km windows.

Section	Metric	PCHIP Turn, 2 km (km)	PCHIP Turn, 3 km (km)	Absolute Shift (km)	Annual Detections, 2 km	Annual Detections, 3 km
LOW	LSI	0.150	0.150	0.000	20/21	20/21
	SHDI	—	—	—	0/21	0/21
	LPI	—	—	—	6/21	6/21
MIDDLE	LSI	0.200	0.200	0.000	21/21	21/21
	SHDI	0.500	0.500	0.000	13/21	13/21
	LPI	—	—	—	10/21	10/21
HIGH	LSI	0.250	0.250	0.000	21/21	21/21
	SHDI	—	—	—	0/21	0/21
	LPI	—	—	—	15/21	15/21

Notes: A dash indicates that no qualifying turning point was identified for the pooled curve within the predefined search range, rather than a zero-valued shift. Annual detections may occur even if the pooled curve has no qualifying turning point. The estimates are descriptive and are not refits of the principal GAM.

**Table A9:** Climate-coefficient estimates from exploratory common-window sensitivity analyses.

Window (km)	Section	Metric	Climate	Beta Per SD	HAC(2) SE	95% Interval	Raw *p*	BH-FDR q
2	LOW	LSI	MeanTemp	0.0757	0.0184	[0.0396, 0.1118]	0.00004	0.00036
2	LOW	LSI	SPEI12	0.0184	0.0190	[−0.0189, 0.0557]	0.33357	0.50036
2	LOW	SHDI	MeanTemp	0.0097	0.0023	[0.0052, 0.0142]	0.00002	0.00036
2	LOW	SHDI	SPEI12	0.0060	0.0015	[0.0031, 0.0090]	0.00006	0.00036
2	LOW	LPI	MeanTemp	−0.2882	0.3820	[−1.0369, 0.4605]	0.45060	0.57934
2	LOW	LPI	SPEI12	0.3885	0.3220	[−0.2426, 1.0197]	0.22763	0.39624
2	MIDDLE	LSI	MeanTemp	−0.1330	0.0873	[−0.3040, 0.0380]	0.12747	0.29279
2	MIDDLE	LSI	SPEI12	0.0572	0.0489	[−0.0386, 0.1530]	0.24215	0.39624
2	MIDDLE	SHDI	MeanTemp	0.0013	0.0030	[−0.0045, 0.0071]	0.65950	0.74194
2	MIDDLE	SHDI	SPEI12	0.0035	0.0013	[0.0009, 0.0062]	0.00891	0.04010
2	MIDDLE	LPI	MeanTemp	0.1859	0.2059	[−0.2177, 0.5895]	0.36660	0.50759
2	MIDDLE	LPI	SPEI12	−0.0416	0.0916	[−0.2212, 0.1380]	0.64979	0.74194
2	HIGH	LSI	MeanTemp	0.1659	0.1096	[−0.0489, 0.3806]	0.13013	0.29279
2	HIGH	LSI	SPEI12	0.1378	0.0884	[−0.0355, 0.3111]	0.11907	0.29279
2	HIGH	SHDI	MeanTemp	−0.0002	0.0009	[−0.0020, 0.0017]	0.86488	0.86488
2	HIGH	SHDI	SPEI12	0.0015	0.0007	[0.0000, 0.0029]	0.04642	0.16711
2	HIGH	LPI	MeanTemp	0.0491	0.2008	[−0.3445, 0.4427]	0.80681	0.85427
2	HIGH	LPI	SPEI12	−0.1844	0.1409	[−0.4606, 0.0919]	0.19083	0.38166
3	LOW	LSI	MeanTemp	0.0906	0.0296	[0.0327, 0.1486]	0.00216	0.01299
3	LOW	LSI	SPEI12	0.0077	0.0218	[−0.0351, 0.0505]	0.72422	0.76682
3	LOW	SHDI	MeanTemp	0.0101	0.0025	[0.0053, 0.0150]	0.00004	0.00036
3	LOW	SHDI	SPEI12	0.0064	0.0015	[0.0033, 0.0094]	0.00004	0.00036
3	LOW	LPI	MeanTemp	−0.4299	0.4155	[−1.2443, 0.3846]	0.30091	0.48215
3	LOW	LPI	SPEI12	0.4273	0.3690	[−0.2960, 1.1505]	0.24694	0.44449
3	MIDDLE	LSI	MeanTemp	−0.1253	0.0895	[−0.3007, 0.0501]	0.16139	0.36312
3	MIDDLE	LSI	SPEI12	0.0573	0.0578	[−0.0560, 0.1707]	0.32143	0.48215
3	MIDDLE	SHDI	MeanTemp	0.0015	0.0029	[−0.0041, 0.0072]	0.59385	0.71928
3	MIDDLE	SHDI	SPEI12	0.0035	0.0013	[0.0009, 0.0061]	0.00850	0.03826
3	MIDDLE	LPI	MeanTemp	0.1720	0.1958	[−0.2117, 0.5557]	0.37956	0.52555
3	MIDDLE	LPI	SPEI12	−0.0444	0.0846	[−0.2103, 0.1214]	0.59940	0.71928
3	HIGH	LSI	MeanTemp	0.1833	0.1126	[−0.0374, 0.4040]	0.10363	0.31088
3	HIGH	LSI	SPEI12	0.1391	0.0912	[−0.0396, 0.3178]	0.12722	0.32714
3	HIGH	SHDI	MeanTemp	−0.0001	0.0010	[−0.0021, 0.0018]	0.88054	0.88054
3	HIGH	SHDI	SPEI12	0.0016	0.0008	[0.0000, 0.0031]	0.04303	0.15492
3	HIGH	LPI	MeanTemp	0.1191	0.2714	[−0.4128, 0.6509]	0.66086	0.74346
3	HIGH	LPI	SPEI12	−0.2306	0.1963	[−0.6153, 0.1541]	0.24006	0.44449

Notes: Contrasts were mean(50–250 m) minus mean(500 m to the indicated window), estimated from 21 annual values with a linear year term and heteroskedasticity/autocorrelation-consistent standard errors (lag 2). Each window includes 18 coefficient tests (3 sections × 3 metrics × 2 climate variables); BH-FDR was applied separately within each window. These are exploratory window comparisons, not the primary M1–M3 models in Section 3.3. Intervals and raw *p*-values are unadjusted for multiplicity.
